# NLRP14 Safeguards Calcium Homeostasis via Regulating the K27 Ubiquitination of Nclx in Oocyte‐to‐Embryo Transition

**DOI:** 10.1002/advs.202301940

**Published:** 2023-07-26

**Authors:** Tie‐Gang Meng, Jia‐Ni Guo, Liu Zhu, Yike Yin, Feng Wang, Zhi‐Ming Han, Lei Lei, Xue‐Shan Ma, Yue Xue, Wei Yue, Xiao‐Qing Nie, Zheng‐Hui Zhao, Hong‐Yong Zhang, Si‐Min Sun, Ying‐Chun Ouyang, Yi Hou, Heide Schatten, Zhenyu Ju, Xiang‐Hong Ou, Zhen‐Bo Wang, Catherine C. L. Wong, Zhonghan Li, Qing‐Yuan Sun

**Affiliations:** ^1^ Fertility Preservation Lab Guangdong‐Hong Kong Metabolism and Reproduction Joint Laboratory Reproductive Medicine Center Guangdong Second Provincial General Hospital Guangzhou 510317 P. R. China; ^2^ State Key Laboratory of Stem Cell and Reproductive Biology Institute of Zoology Chinese Academy of Sciences Beijing 100101 P. R. China; ^3^ School of Basic Medical Sciences Peking University Health Science Center Beijing 100191 P. R. China; ^4^ Center for Growth Metabolism & Aging Key Laboratory of Bio‐Resource and Eco‐Environment of Ministry of Education College of Life Sciences Sichuan University Chengdu 610017 P. R. China; ^5^ Department of Histology and Embryology Harbin Medical University Harbin 150088 P. R. China; ^6^ Department of Veterinary Pathobiology University of Missouri Columbia MO 65211 USA; ^7^ Key Laboratory of Regenerative Medicine of Ministry of Education Institute of Aging and Regenerative Medicine Jinan University Guangzhou Guangdong 510632 P. R. China; ^8^ Department of Medical Research Center State Key Laboratory of Complex Severe and Rare Diseases Peking Union Medical College Hospital Chinese Academy of Medical Science & Peking Union Medical College Beijing 100730 P. R. China; ^9^ Tsinghua University‐Peking University Joint Center for Life Sciences Tsinghua University Beijing 100084 P. R. China

**Keywords:** adenosine triphosphate (ATP), calcium homeostasis, early embryonic development, maternal effect genes, mitochondria, NCLX, NLRP14, UHRF1

## Abstract

Sperm‐induced Ca^2+^ rise is critical for driving oocyte activation and subsequent embryonic development, but little is known about how lasting Ca^2+^ oscillations are regulated. Here it is shown that NLRP14, a maternal effect factor, is essential for keeping Ca^2+^ oscillations and early embryonic development. Few embryos lacking maternal NLRP14 can develop beyond the 2‐cell stage. The impaired developmental potential of *Nlrp14*‐deficient oocytes is mainly caused by disrupted cytoplasmic function and calcium homeostasis due to altered mitochondrial distribution, morphology, and activity since the calcium oscillations and development of *Nlrp14*‐deficient oocytes can be rescued by substitution of whole cytoplasm by spindle transfer. Proteomics analysis reveal that cytoplasmic UHRF1 (ubiquitin‐like, containing PHD and RING finger domains 1) is significantly decreased in *Nlrp14*‐deficient oocytes, and *Uhrf1*‐deficient oocytes also show disrupted calcium homeostasis and developmental arrest. Strikingly, it is found that the mitochondrial Na^+^/Ca^2+^ exchanger (NCLX) encoded by *Slc8b1* is significantly decreased in the *Nlrp14^mNull^* oocyte. Mechanistically, NLRP14 interacts with the NCLX intrinsically disordered regions (IDRs) domain and maintain its stability by regulating the K27‐linked ubiquitination. Thus, the study reveals NLRP14 as a crucial player in calcium homeostasis that is important for early embryonic development.

## Graphical abstract

The authors show that calcium homeostasis including Ca^2+^ concentration dynamics, mitochondria dynamics, and ATP level are affected in *Nlrp14*‐deficient oocytes as well as in *Uhrf1*‐deficient oocytes during oocyte‐to‐embryo transition. Moreover, they demonstrate that NLRP14 interacts with the NCLX IDRs domain and maintains its stability by regulating the K27‐linked ubiquitination.

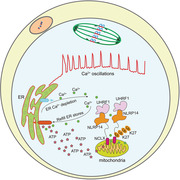

## 1 Introduction

The initiation events of early embryonic development in mammals are mainly controlled by maternal effectors which are encoded by maternal effect genes and accumulated during oogenesis. However, our understanding of the function of maternal effect genes in early embryo development is very limited. Maternal mRNAs and proteins are accumulated during oocyte growth, which is required for subsequent fertilization and early embryo development.^[^ ^1^ ^]^ A growing body of evidence has supported a role for maternal factors, such as MATER (NLRP5), PADI6, and STELLA (also named DPPA3 or PGC7), in early embryonic development.^[^ ^2^ ^]^ The NLRP (Nucleotide‐binding oligomerization domain, Leucine‐rich Repeat, and Pyrin domain containing) family is composed of 14 members containing similar structures. Among this family, NLRP1 and NLRP3 are involved in apoptosis and inflammation. However, several members (including NLRP2, 4, 5, 6, 9, 14) are reproduction‐related. *Nlrp5* (also known as *Mater*) null female mice are infertile due to 2‐cell arrest.^[^ ^2a^ ^]^ Decreased expression of *Mater* and *Nlrp14* has been observed in oocytes during maternal aging.^[^ ^3^ ^]^ The phylogenetic tree of NLR LRR domains analysis has shown that NLRP14 and MATER are orthologues within a subgroup.^[^ ^4^ ^]^ However, the functions of maternal NLRP14 in early development remain unknown.

Sperm‐induced oocyte activation is a critical starting step for a new life, and oocyte activation inefficiency is the most challenging problem for failed fertilization and embryonic development. Upon fertilization, Ca^2+^ is released from the intracellular Ca^2+^ stores, followed by a series of repetitive Ca^2+^ transients (hereafter, Ca^2+^ oscillations) that persist for several hours in oocytes.^[^ ^5^ ^]^ Mitochondria in oocytes provide ATP to participate in the continuous transport of Ca^2+^ into the ER, maintain low cytoplasmic Ca^2+^ concentration ([Ca^2+^]i), and maintain long‐term [Ca^2+^]i oscillations after oocyte activation.^[^ ^6^ ^]^ At the same time, mitochondria also uptake cytoplasmic Ca^2+^, which regulates the metabolism of mitochondria.^[^ ^7^ ^]^ Mitochondrial Ca^2+^ homeostasis regulates oxidative phosphorylation by affecting cell energy supply.^[^ ^7^ ^]^ However, little is known about how Ca^2+^ oscillations are regulated and maintained during oocyte activation until now.

In somatic cells, nuclear localization of UHRF1 recognizes hemimethylated DNA and then recruits DNMT1 to the replication foci to maintain DNA methylation.^[^ ^8^ ^]^ Li et al. have demonstrated that STELLA prevented DNA methylation mediated by DNMT1 by regulating the localization of UHRF1 in oocytes.^[^ ^9^ ^]^ Very recently, it has been showed that DNMT1 and its cofactor UHRF1 changed from cytoplasmic localization to nuclear localization in zygotes in the absence of *Nlrp14*, which impaired passive DNA demethylation and zygotic genome activation.^[^ ^10^ ^]^ However, UHRF1 showed almost entirely cytoplasmic localization in oocytes,^[^ ^9^, ^11^ ^]^ and the exclusive function of cytoplasmic NLRP14 in oocytes remains unknown.

In the present study, we generated *Nlrp14* knockout mice using CRISPR/Cas9 and found that *Nlrp14*‐null oocytes showed development arrest. Through systematically analyzing the quality indicators of oocytes and early embryos, proteomic analysis, spindle transfer, and a series of biochemical experiments, we showed that infertility and early embryonic development failure of *Nlrp14^mNull^* female mice are mainly due to impaired cytoplasmic Ca^2+^ homeostasis. NLRP14 interacts with NCLX to regulate NCLX stability via K27‐linked ubiquitination, revealing the novel function of maternal NLRP14 in maintaining calcium homeostasis in early development.

## 2 Results

### 2.1 *Nlrp14* is a Maternal Effect Gene Required for Early Embryonic Development in Mice

**Figure 1:**
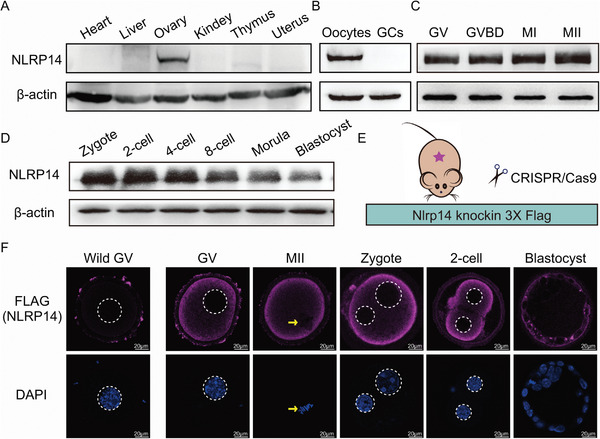
Developmental expression of NLRP14 in mouse. A) Western blot analysis showed that NLRP14 is only expressed in the ovaries rather than other tissues including heart, liver, kidney, thymus, and uterus in female mice. B) Western blot showed that NLRP14 was only expressed in oocytes rather than in granulosa cells (GCs). C) The expression pattern of NLRP14 during oocyte maturation. D) The expression pattern of NLRP14 during early embryonic development. E) Establishment of Nlrp14‐3xflag knock‐in mouse model, the tags including *3xflag* were inserted before stop codon of *Nlrp14* CDS using Crispr/Cas9. F) Representative images of subcellular localization of NLRP14 during oocyte maturation and early embryonic development. The oocytes and embryos, derived from Nlrp14‐3xflag knock‐in female mice, were immunolabeled with FLAG antibody (pink) and counterstained with DAPI (blue). Scale bar, 20 µm.

We first analyzed the expression profile of *Nlrp14*. Western blots showed that NLRP14 was predominantly expressed in mouse ovaries, but not in other tissues in female mice (**Figure** **1**A). Moreover, the NLRP14 protein was primarily detected in oocytes rather than in granulosa cells (Figure 1B). In mouse oocytes and preimplantation embryos, NLRP14 protein remained at high levels until the blastocyst stage (Figure 1C,D). Quantitative RT‐PCR (qRT‐PCR) analysis showed that *Nlrp14* mRNA was highly expressed in oocytes and persisted until 2‐cell stage embryos but significantly decreased after this stage (Figure S1A, Supporting Information). Thus, the expression *Nlrp14* showed a typical pattern of maternal effect genes. In order to accurately detect the localization of NLRP14 in oocytes and early embryos, we constructed *Nlrp14‐3xflag* knock‐in mice (Figure 1E and Figure S1B, Supporting Information). Immunofluorescence showed that NLRP14 localized exclusively to the cytoplasm in oocytes and preimplantation embryos (Figure 1F and Figure S1C,D, Supporting Information). These findings indicate that *Nlrp14* is a maternal effect gene.

**Figure 2:**
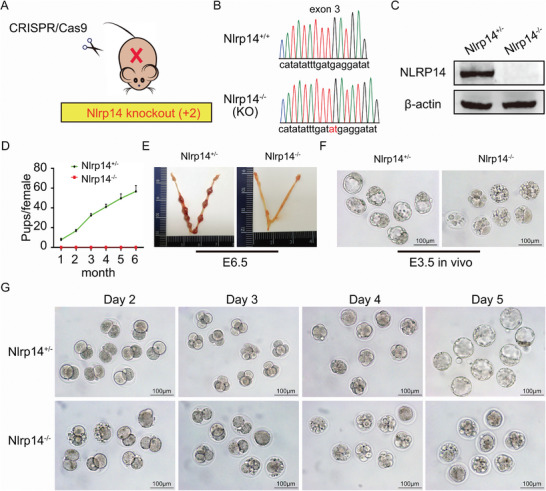
*Nlrp14* is a maternal effect gene required for early embryonic development in mice. A,B) Establishment of Nlrp14 knockout mouse model carrying frameshift mutations (inserted two nucleotides in exon3). C) Western blot analysis of protein level in *Nlrp14^+/−^* and *Nlrp14^−/−^* oocytes. Level of β‐actin was used as an internal control. D) Breeding assays showed complete infertility of the *Nlrp14^−/−^* female mice. Continuous breeding assessment showed the cumulative number of progeny per female *Nlrp14^+/−^* and *Nlrp14^−/−^* mouse for 6 months. At least six mice of each genotype were used. Data are the mean ± SEM (*n* = 6). E) Representative uterus and number of implantation sites at E6.5 in *Nlrp14^+/−^* and *Nlrp14^−/−^* mice. F) Both *Nlrp14^+/−^* and *Nlrp14^−/−^* female mice underwent natural ovulation after mating with WT male mice; embryo development was examined in the uterus at day E3.5. G) Representative images of embryos from *Nlrp14^+/−^* and *Nlrp14^−/−^* females cultured in KSOM medium at Day 2, Day 3, Day 4, and Day 5, respectively. Scale bar, 100 µm.

To investigate the physiological function of *Nlrp14*, we established a line of *Nlrp14* knockout mice using the CRISPR/Cas9 system. Mice carrying frameshift mutations (inserted two nucleotides in exon3) in *Nlrp14* allele were obtained (**Figure** **2**A,B). The genotypes of mice including *Nlrp14^+/+^*, *Nlrp14^+/−^* and *Nlrp14^−/−^* were confirmed by DNA sequencing. Subsequent germ‐line transmission was obtained and null mice were generated within a mixed genetic CD1 background. Western blots showed that NLRP14 protein completely disappeared in *Nlrp14^−/−^* (hereafter, *Nlrp14^mNull^*) oocytes (Figure 2C).

*Nlrp14^−/−^* mice appeared normal at birth and had no discernible histologic abnormalities. By natural mating, we found that the *Nlrp14^mNull^* female mice were infertile, while the *Nlrp14^+/+^*, *Nlrp14^+/−^* (hereafter, control) mice showed normal fertility (Figure 2D). To find the reason for female infertility, we analyzed the uterus at post‐implantation stages and found no embryos (Figure 2E). These results clearly showed that loss of the maternal NLRP14 caused early embryonic lethality. At E3.5, blastocysts were flushed from the uterus of control mice but not from *Nlrp14^mNull^* mice (Figure 2F). Then, we isolated zygotes from control and *Nlrp14^mNull^* females after mating with normal males. The infertility of *Nlrp14^mNull^* female mice appeared not to be related to ovulation since approximately the same number of zygotes could be obtained from *Nlrp14^mNull^* mice when compared with control mice in natural ovulation assays. However, when culturing these embryos in vitro, few embryos derived from *Nlrp14^mNull^* mice developed beyond the 2‐cell stage while control embryos developed into morula and blastocyst at E2.5 and E3.5, respectively (Figure 2G). Interestingly, some oocytes that appeared to be unfertilized are actually penetrated by sperm, but the chromatin of sperm and oocytes were unable to complete de‐condensation to form a pronucleus (Figure S1E, Supporting Information). In addition, the *Nlrp14^mNull^* mice were still infertile, even within a mixed genetic CD1 background, implying an indispensable function of NLRP14. Collectively, the above analysis shows that maternal NLRP14 is required for fertilization and early embryonic development.

### 2.2 Maternal NLRP14 Regulates the Stability and Subcellular Distribution of UHRF1

**Figure 3:**
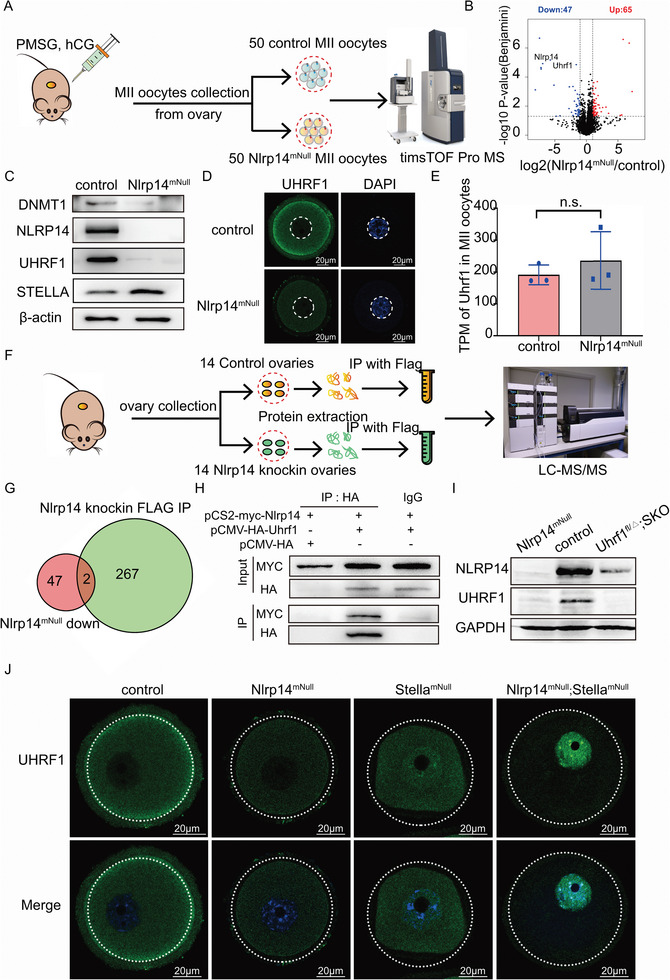
Maternal NLRP14 and UHRF1 form heteromeric complexes A) Schematic of MII oocytes collection and timTOF Pro MS analysis. MS samples from three independent experiments were used for Mass spectrometry analysis. B) Significantly upregulated (red) and downregulated (blue) proteins in *Nlrp14^mNull^* oocytes. C) Immunoblotting analyses of the control and *Nlrp14^mNull^* MII oocytes were performed using antibodies against the indicated proteins. D) The signal of UHRF1 in control and *Nlrp14^mNull^* oocytes. Scale bar, 20 µm. E) Average expression of *Uhrf1* mRNA during oocyte maturation and early embryonic development. Analysis is based on our RNA‐seq data. Data are the mean ± SEM (*n* = 3). F) Schematic of immunoprecipitation coupled Mass Spectrometry analysis for NLRP14 interacting proteins. IP samples from three independent experiments were used for Mass spectrometry analysis. G) Venn diagram depicting common proteins identified from down‐regulated proteins in *Nlrp14^mNull^* oocytes and NLRP14 interacting proteins. H) Interaction between NLRP14 and UHRF1 was confirmed by immunoprecipitation. HA‐tag, HA‐tagged mouse UHRF1 and myc‐tagged mouse NLRP14 were expressed in HEK293T cells as indicated for 48 h, and then Co‐IP (HA‐MYC) and Western blot analysis for UHRF1 and NLRP14. I) Immunoblotting analyses of the control and *Nlrp14^mNull^* MII oocytes were performed using antibodies against the indicated proteins. J) The signal of UHRF1 in control, *Nlrp14^mNull^* and *Nlrp14^mNull^;Stella^mNull^* oocytes, respectively. Scale bar, 20 µm.

To find the causes for embryonic development arrest in *Nlrp14^mNull^* mice, we collected 50 control MII oocytes and 50 *Nlrp14^mNull^* MII oocytes (three biological replicates in each group) for quantitative proteomic analyses using an advanced trapped‐ion mobility selecting (timsTOF Pro) mass spectrometer (**Figure** **3**A, Table S1, Supporting Information). Subsequent quantitative proteomic analysis showed that 3509 proteins were quantified and that a total of 112 proteins were differently expressed, including 65 upregulated proteins and 47 downregulated proteins in *Nlrp14^mNull^* oocytes compared with control oocytes (Figure 3B). Strikingly, we found that the UHRF1 was significantly decreased in the *Nlrp14^mNull^* group as well as the DNMT1. Western blot and immunofluorescent detection also confirmed this result (Figure 3C,D). It should be pointed out that embryos derived from *Dnmt1*‐deficient oocytes showed normal preimplantation development.^[^ ^12^ ^]^ Notably, maternal ablation of UHRF1 caused a similar phenotype with *Nlrp14^mNull^* embryos; both showed 2‐cell arrest.^[^ ^11^, ^13^ ^]^ Interestingly, the STELLA expression level even increased partially in *Nlrp14^mNull^* oocytes. However, there was no significant difference in the *Uhrf1* mRNA levels between *Nlrp14^mNull^* oocytes and control oocytes (Figure 3E). To unequivocally examine the proteins directly affected by maternal NLRP14, we simultaneously conducted immunoprecipitation coupled to mass spectrometry (IP–MS) using *Nlrp14‐3xflag* knock‐in mice. We identified 267 proteins that specifically interact with NLRP14 within mouse oocytes (Figure 3F and Table S2, Supporting Information). Among the proteins identified in IP‐MS, only UHRF1 and NLRP14 itself were observed in downregulated protein sets in *Nlrp14^mNull^* oocytes (Figure 3G). Interaction between NLRP14 and UHRF1 was also confirmed by immunoprecipitation, indicating that NLRP14 and UHRF1 could form heteromeric complexes (Figure 3H and Figure S2, Supporting Information). Significantly, the NLRP14 was obviously decreased in oocytes lacking maternal UHRF1 (Figure 3I), suggesting the stability of one protein is dependent on the presence of the other. Interestingly, unlike in *Stella^mNull^* fully grown oocytes (FGOs) where a large proportion of UHRF1 became cytoplasmic distribution, *Nlrp14^mNull^; Stella^mNull^* FGOs showed exclusive nuclear distribution of residual UHRF1 (Figure 3J).

### 2.3 Abnormal Calcium Homeostasis in *Nlrp14^mNull^* Oocytes

To clarify the reason for early embryonic development arrest, we systematically analyzed the quality indicators of oocytes and early embryos. Loss of maternal NLRP14 had no obvious influence on meiotic maturation as the *Nlrp14^mNull^* oocytes showed normal polar body extrusion (PBE) rates and normal spindle morphology (Figure S3A, Supporting Information). To analyze aneuploidy in metaphase‐II oocytes, we employed chromosome spreading of metaphase‐II oocytes and counted chromosomes (Figure S3B, Supporting Information). There was no difference in the number of chromosomes between control oocytes and *Nlrp14^mNull^* oocytes. Cortical granules (CGs) are membrane‐bound secretory organelles located at the cortex of MII oocytes, and formation of the cortical granule‐free domain (CGFD) is not only a criterion of cytoplasmic maturation but also a feature of oocyte polarization. Similar to the control MII oocytes, obvious CGFD formed in *Nlrp14^mNull^* oocytes (Figure S3C, Supporting Information). Then we performed RNA sequencing (RNA‐seq) with MII oocytes to investigate the effects of maternal *Nlrp14* deletion on the transcriptome profile in oocytes and found that there were very high correlation coefficients in expression profiles between *Nlrp14^mNull^* oocytes and control oocytes (Figure S4A, Supporting Information). Moreover, we analyzed the decay of representative maternal mRNA in control and *Nlrp14^mNull^* oocytes during meiotic maturation and after fertilization by qRT–PCR. These maternal mRNAs were degraded in MII oocytes and 2‐cell embryos in *Nlrp14^mNull^* mice, similar to the control (Figure S4B, Supporting Information). A subset of “maternal effect genes”, namely *Mater* (*Nlrp5*), *Tle6*, *Ooep*, *Filia* (*Khdc3*), and *Padi6*, encode proteins of the subcortical maternal complex (SCMC). Recently, a subcortical maternal complex (SCMC) was identified to be essential for mouse preimplantation development, which was composed of multiple proteins encoded by several maternal effect genes (*Mater*, *Tle6*, *Ooep*, *Filia*, and *Padi6*). Thus, we asked whether maternally expressed NLRP14 linked SCMC to function in early embryonic development. However, there was no difference in the expression levels of SCMC proteins and mRNA between control and *Nlrp14^mNull^* oocytes (Figure S4C,D, Supporting Information).

**Figure 4:**
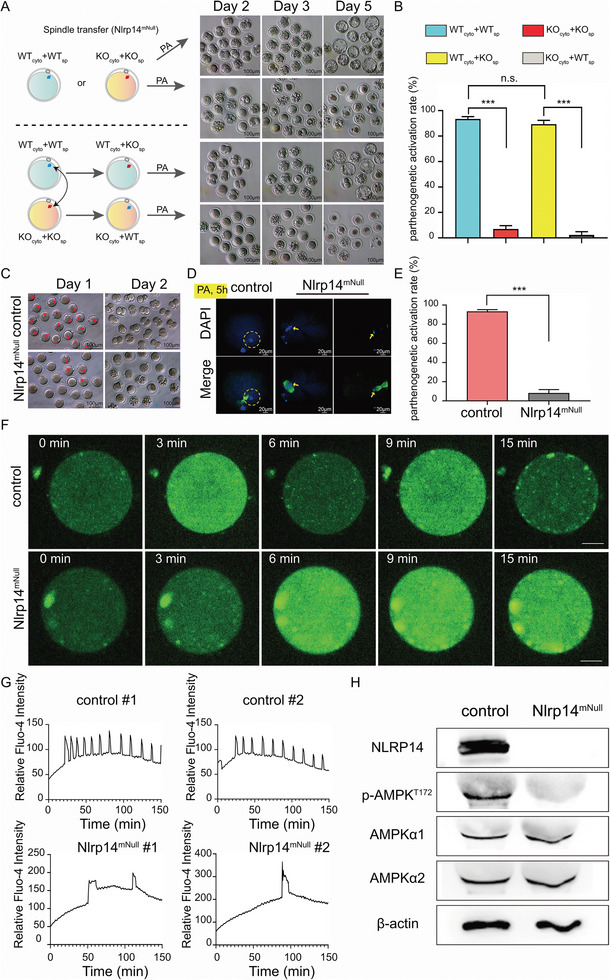
Ablation of maternal NLRP14 caused the failure of the [Ca^2+^]i induced by parthenogenetic activation. A) A schematic illustration of the spindle transfer assay between control and *Nlrp14^mNull^* MII oocytes. WT indicates control (*Nlrp14^+/−^*), KO indicates *Nlrp14^mNull^*, PA indicates parthenogenetic activation. The hybrid oocytes produced by spindle exchange were parthenogenetically activated in an activation medium for 6 h, then cultured in KSOM. Representative images of parthenogenetically activated embryos with the indicated genotypes at day 2, day 3, and day 5. B) Bar charts showing percentages of parthenogenetic activation with indicated genotypes and treatments. Data are the mean ± SEM (*n* = 3). ***p <0.001. C) control and *Nlrp14^mNull^* MII oocytes were parthenogenetically activated in an activation medium for 6 h, then cultured in KSOM. Representative images of parthenogenetic activated embryos with the indicated genotypes at day 1 and day 2, respectively. Red arrowheads show visible pronuclei. D) Representative images of immunostaining for DNA (blue) and α‐tubulin (green) showing the MII exit and pronuclei formation in the parthenogenetic‐activated embryos. Scale bar, 20 µm. E) Bar charts showing percentages of parthenogenetic activation with indicated genotypes. ***p <0.001. F) Fluo‐4‐AM staining of oocytes showed the intracellular Ca^2+^ concentration dynamics at different stages of two continuous [Ca^2+^]i oscillations during PA. The oocytes are indicated with genotypes. Scale bar, 20 µm. G) [Ca^2+^]i oscillation patterns after parthenogenetic activation of oocytes indicated with genotypes, respectively. H, Western blot analysis of the energy sensor AMPK using the indicated antibodies in control and *Nlrp14^mNull^* oocytes. AMPK is composed of three subunits, the α subunit has catalytic activity (including two or three isoforms (α1 and α2)), its Thr172 phosphorylation is the target for regulating the catalytic activity of the enzyme, while the β and γ subunits are regulatory subunits. The experiment was repeated three times independently.

Next, to determine whether *Nlrp14^mNull^* embryonic arrest is caused by cytoplasmic defects, we reciprocally exchanged the spindle–chromosome complex between control and *Nlrp14^mNull^* MII oocytes, then carried out parthenogenetic activation (PA) and further culture of the reconstructed oocytes in KSOM medium (**Figure** **4**A). Obviously, the reconstructed MII oocytes composed of control cytoplasm and *Nlrp14^mNull^* chromosomes (termed WT_cyto_+KO_sp_) could develop beyond the 2‐cell stage, similar to WT_cyto_+WT_sp_ MII oocytes upon parthenogenetic activation. On the contrary, the reconstructed oocytes composed of *Nlrp14^mNull^* cytoplasm and control chromatin (termed KO_cyto_+WT_sp_) showed activation inefficiency after parthenogenetic activation (Figure 4B). Meanwhile, the WT_cyto_+KO_sp_ MII oocytes showed a relatively normal calcium oscillation pattern, while KO_cyto_+WT_sp_ MII oocytes displayed a consistently high intracellular Ca^2+^ concentration upon PA (Figure S5 and Videos S1 and S2, Supporting Information). We next examined the developmental potential of *Nlrp14^mNull^* oocytes after parthenogenetic activation. Surprisingly, the *Nlrp14^mNull^* oocytes rarely formed a pronucleus after parthenogenetic activation, showing severe parthenogenetic activation defects, indicated by MII phase exit failure and chromatin de‐condensation failure (Figure 4C,D). In addition, when parthenogenetically activated *Nlrp14^mNull^* oocytes were cultured in KSOM until the next day, few reached the 2‐cell stage, accompanied by a large percentage of deaths (Figure 4E). We then examined cytoplasmic Ca^2+^ concentrations in control oocytes and *Nlrp14^mNull^* oocytes during parthenogenetic activation. We used Fluo‐4‐AM, an improved version of the calcium indicator, to measure the intracellular calcium level. The control oocytes showed normal Ca^2+^ oscillation patterns; the initial Ca^2+^ wave was followed by repetitive [Ca^2+^]i transients. However, the *Nlrp14^mNull^* oocytes maintained a high cytoplasmic Ca^2+^ concentration and there were no [Ca^2+^]i oscillations, implying that Ca^2+^ homeostasis was defective in oocytes lacking maternal NLRP14 (Figure 4F,G and Videos S3 and S4, Supporting Information). In addition, AMP‐activated protein kinase (AMPK) is an important energy sensor to sense energy deficiency, which could be activated in a CAMKK2‐dependent manner. Western blotting showed that the activity of AMPK (Thr172) was significantly reduced in *Nlrp14^mNull^* oocytes, implying that prolonged continuous (non‐oscillatory) elevation of [Ca^2+^]i reduced the CaMKK2 activity (Figure 4H). Collectively, these results show that the low quality of *Nlrp14^mNull^* oocytes is mainly due to cytoplasmic defects and disrupted calcium homeostasis.

### 2.4 Loss of NLRP14 Impairs Mitochondria Dynamics and Functions

**Figure 5:**
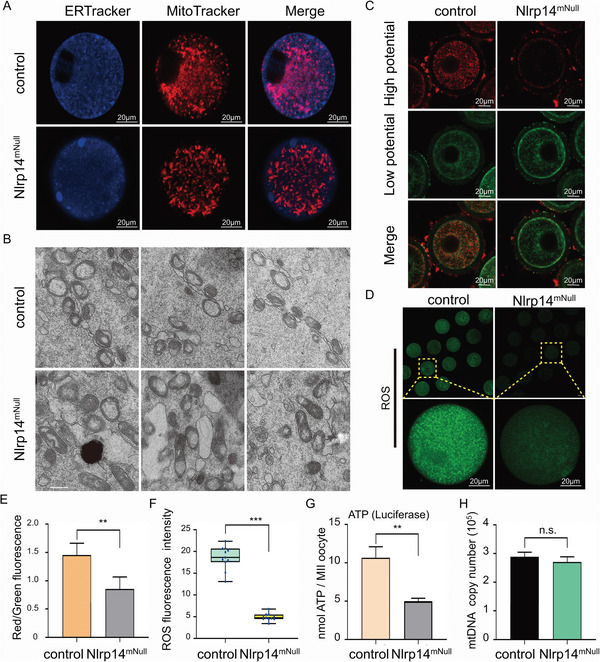
Abnormal mitochondrial morphology and mitochondrial activity in *Nlrp14^mNull^* oocytes. A) ERs and mitochondria were labeled with ER‐Tracker (blue) and MitoTracker (red) in control and *Nlrp14^mNull^* oocytes. Scale bar, 20 µm. B) Electron micrographs of 6‐week‐old control and *Nlrp14^mNull^* oocytes. White arrows indicate the mitochondria in oocytes with the indicated genotypes. Scale bar, 500 nm. C) Distribution of mitochondria with high membrane potential (red) and low membrane potential (green) in oocytes with the indicated genotypes, respectively. Scale bar, 20 µm. D) Representative images of ROS fluorescence of MII oocytes with the indicated genotypes, respectively. Images were analyzed by confocal microscopy with identical fluorescence parameters. Scale bar, 20 µm. E), Relative fluorescence intensity of ratio of red/green fluorescence analysis for each oocyte was conducted using Image J software. Significant difference between control and *Nlrp14^mNull^* oocytes was observed. Data are expressed as mean±SEM of at least three independent experiments. **p <0.01. Data are the mean ± SEM (*n* = 20). F) Quantitative analysis of ROS fluorescence intensity. The fluorescence intensity analysis for each oocyte was conducted using Image J software. Data are expressed as mean±SEM of at least three independent experiments. ***p <0.001. Data are the mean ± SEM (*n* = 20). G) The adenosine triphosphate (ATP) content of mouse oocytes with the indicated genotypes, respectively. Data are the mean ± SEM (*n* = 60). ATP was measured using a Berthold Lumat LB 9501 luminometer and a commercial assay kit. Data are expressed as mean±SEM of at least three independent experiments. **p <0.01. H, mtDNA copy numbers of MII oocytes in all three groups were analyzed by RT‐qPCR. Data from more than 20 MII oocytes were analyzed for each group. Data are expressed as mean±SEM of at least three independent experiments (*n* = 3). n.s. represents the non‐significant difference.

We next sought to find the cause of disrupted calcium homeostasis. We examined organelle distribution in control oocytes and *Nlrp14^mNull^* oocytes using ER‐Tracker and Mito‐Tracker to label the ER and mitochondria, respectively (**Figure** **5**A). Strikingly, altered mitochondrial morphology was observed in *Nlrp14^mNull^* oocytes. The major axis of mitochondria in *Nlrp14^mNull^* oocytes became longer compared to the mitochondria in the control oocytes. Electron microscopy (EM) observation also showed that mitochondria in *Nlrp14^mNull^* oocytes were elongated, suggesting that loss of maternal NLRP14 severely affected mitochondrial morphology (Figure 5B and Figure S6, Supporting Information). One particular note is that mitochondria are numerous, small, and round in appearance in oocytes when compared to somatic cells. Mitochondrial dynamics are essential for mitochondrial energy metabolism and stress response. Inconsistent with the radial mitochondria distribution around the nucleus in control oocytes before GVBD, the mitochondria were always concentrated in the subcortical region in *Nlrp14^mNull^* oocytes, implying defects in mitochondria dynamics (Videos S5–S8, Supporting Information). Meanwhile, normal mitochondrial membrane potential (MMP) is a prerequisite for mitochondria to carry out respiratory activity and produce ATP, which is necessary for the maintenance of mitochondrial function. Mitochondrial membrane potential (Δψ) was assessed by JC‐1 staining. Surprisingly, compared to the rounded mitochondria in control oocytes, the elongated mitochondria in *Nlrp14^mNull^* oocytes showed remarkably reduced MMP, suggesting compromised mitochondrial activity (Figure 5C,E). The cellular reactive oxygen species (ROS) level is proportional to the activity of mitochondrial electron transport. Consistent with the compromised mitochondrial activity, we found that the ROS level was obviously reduced in *Nlrp14^mNull^* oocytes (Figure 5D,F). Similarly, ATP content analyzed by luminometric analysis was significantly decreased in *Nlrp14^mNull^* oocytes (Figure 5G). Subsequently, we tested whether the mtDNA copy number was affected in *Nlrp14^mNull^* oocytes using RT‐qPCR, and showed that there was no difference in the mtDNA copy number between *Nlrp14^mNull^* oocytes and control oocytes (Figure 5H). Taken together, the above findings indicated that maternal NLRP14 is essential for mitochondrial energy metabolism, which is closely associated with Ca^2+^ homeostasis in mouse oocytes.

### 2.5 Decreased UHRF1 Expression in *Nlrp14^mNull^* Oocytes is also a cause for Disrupted Calcium Homeostasis

**Figure 6:**
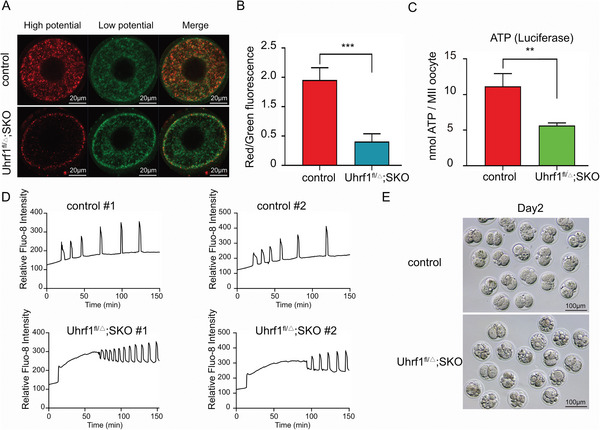
UHRF1 is essential for maintaining calcium homeostasis in oocytes. A) Distribution of mitochondria with high membrane potential (red) and low membrane potential (green) in oocytes with the indicated genotypes, respectively. Scale bar, 20 µm. B) Relative fluorescence intensity of ratio of red/green fluorescence analysis for each oocyte was conducted using Image J software. Significant difference between control and *Uhrf1^fl/△^;SKO* oocytes was observed. Data are expressed asmean±SEM of at least three independent experiments (*n* = 3). **p <0.001. C) The adenosine triphosphate (ATP) content of mouse oocytes with the indicated genotypes, respectively. ATP was measured using a Berthold Lumat LB 9501 luminometer and a commercial assay kit. Data are expressed as mean±SEM of at least three independent experiments(n = 3). **p <0.01. D) [Ca^2+^]i oscillation patterns after parthenogenetic activation of oocytes with indicated genotypes, respectively. E) control and *Uhr^f1fl/△^;SKO* MII oocytes were parthenogenetically activated in an activation medium for 6 h, then cultured in KSOM. Representative images of parthenogenetic activated embryos with the indicated genotypes at day 2, respectively.

Given that NLRP14 and UHRF1 formed heteromeric complexes and were highly expressed in mouse oocytes, and that deletion of NLRP14 decreased UHRF1 expression, we hypothesized that loss of maternal UHRF1 also affected mitochondrial function and Ca^2+^ homeostasis. The *Uhrf1*‐deficient oocytes showed a lower MMP in contrast to control oocytes, demonstrating that UHRF1 played a vital role in maintaining normal mitochondrial function (Figure 6A,B). Consistent with in *Nlrp14^mNull^* oocytes, the ATP content was significantly decreased in *Uhrf1^fl/△^;SKO* oocytes (Figure 6C). Furthermore, *Uhrf1*‐deficient oocytes displayed aberrant [Ca^2+^]i oscillations, which maintained a high intracellular Ca^2+^ concentration after Ca^2+^ release from intracellular stores (Figure 6D and Videos S9 and S11, Supporting Information) and showed severe parthenogenetic activation defects (Figure 6E). Taken together, our results indicate that maternal UHRF1 also plays a vital role in safeguarding the Ca^2+^ homeostasis in mouse oocytes rather than only causing the aberrant DNA methylome in oocytes lacking maternal STELLA.

### 2.6 Maternal Depletion of NLRP14 Leads to Loss of NCLX and Impairs Mitochondrial Ca^2+^ Dynamics

**Figure 7:**
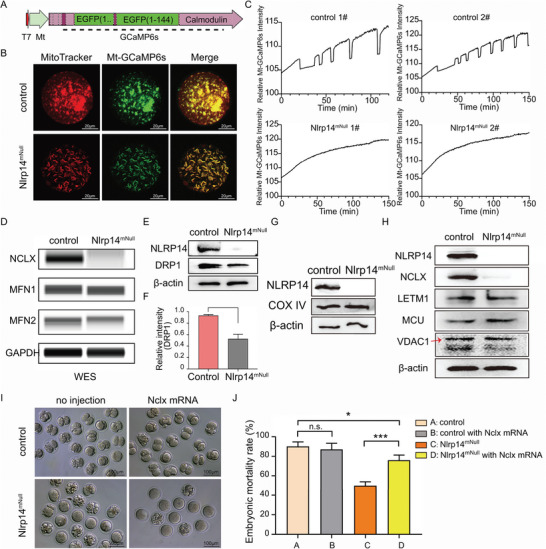
Maternal NLRP14 mainly affected Ca^2+^ homeostasis by regulating the stability of NCLX in mouse oocytes. A) Schematic representation of Mt‐GCaMP6s. Ca^2+^ sensors GCaMP6s were fused with mitochondrial localization signal peptide under the control of T7 promoter. B) Representative images of control and *Nlrp14^mNull^* MII oocytes, which were microinjected with *Mt‐GCaMP6s* mRNA (green) and labeled with MitoTracker (red). Mt‐GCaMP6s was colocalized with MitoTracker. Scale bar, 20 µm. C) [Ca^2+^]m oscillation patterns after parthenogenetic activation of MII oocytes with indicated genotypes, respectively. D) Capillary‐based immunoassays for indicated proteins in oocytes with indicated genotypes, respectively. Loading control, GAPDH. E) Immunoblotting analyses of the control and *Nlrp14^mNull^* MII oocytes were performed using antibodies against the indicated proteins. F) Bar charts showing level of DRP1 in MII oocytes with indicated genotypes. G) Mitochondrial content was evaluated by COX IV. Western blots showing similar mitochondrial components between control and *Nlrp14^mNull^* MII oocytes. H) Immunoblotting analyses of the control and *Nlrp14^mNull^* MII oocytes were performed using antibodies against the indicated proteins. I) Both control and *Nlrp14^mNull^* MII oocytes were microinjected with *Nclx* mRNA, respectively. After culturing for 2 h, these oocytes were parthenogenetically activated in an activation medium for 6 h, then cultured in KSOM. Representative images of parthenogenetic activated embryos with indicated genotypes at day 2, respectively. Scale bar, 100 µm. J), Bar charts showing percentages of parthenogenetic‐activated embryonic mortality with indicated genotypes and treatments. Data are the mean ± SEM (*n* = 3).

Mitochondria sequester and release of Ca^2+^ affects the pattern of [Ca^2+^]i in the cytosol, which acts as Ca^2+^ buffer. Mitochondrial Ca^2+^ activates key enzymes involved in ATP synthesis at the electron transport chain (ETC). To monitor mitochondrial Ca^2+^ concentration ([Ca^2+^]m) dynamic changes during PA, we employed a genetically‐encoded Ca^2+^ indicator Mt‐GCaMP6s (Ca^2+^ sensors GCaMP6s was fused with mitochondrial localization signal peptide cloned from Trmt10c gene) (**Figure** **7**A). As shown in Figure 7B, Mt‐GCaMP6s was colocalized with mitochondria. Strikingly, [Ca^2+^]m dynamic changes completely disappeared in *Nlrp14^mNull^* oocytes, implying impaired [Ca^2+^]m homeostasis in oocytes lacking maternal NLRP14 (Figure 7C and Videos S12 and S13, Supporting Information). [Ca^2+^]m homeostasis is mediated by controlling the expression and activity of mitochondrial Ca^2+^ channels and transporters, which are required for the uptake and extrusion of mitochondrial Ca^2+^. Naturally, we asked which Ca^2+^ channels and transporters were affected in *Nlrp14^mNull^* oocytes. Control oocytes and *Nlrp14^mNull^* oocytes were collected for Western blot analysis. Surprisingly, despite no difference in the mitochondrial content, the expression level of NCLX (the mitochondrial Na^+^/Ca^2+^/Li^+^ exchanger, also known as SLC8B1) was severely reduced in mitochondria‐associated Ca^2+^ channels in *Nlrp14^mNull^* oocytes compared with control oocytes (Figure 7G,H). In addition, the elongated mitochondria in *Nlrp14^mNull^* oocytes might not be caused by changes in mitochondrial fusion‐related proteins MFN1 and MFN2 (Figure 7D). Also, the protein level of mitochondrial fission‐related protein DRP1 was slightly decreased in *Nlrp14^mNull^* oocytes (Figure 7E,F). Then we asked whether supplementation of exogenous *Nclx* mRNA could rescue the phenotype of parthenogenetic activation failure of *Nlrp14^mNull^* oocytes. Although the *Nlrp14^mNull^* oocytes supplemented with exogenous *Nclx* mRNA still could not reach the 2‐cell stage, the embryonic mortality rate was significantly decreased for *Nlrp14^mNull^* oocytes microinjected with *Nclx* mRNA (Figure 7I,J). Similarly, treatment of control oocytes with NCLX inhibitor, CGP37157, during parthenogenetic activation led to oocyte death (Figure S7, Supporting Information). The above results suggest that maternal NLRP14 is required for [Ca^2+^]m homeostasis by regulating NCLX level.

### 2.7 NLRP14 Maintains the Stability of NCLX by Regulating its K27 Ubiquitination

**Figure 8:**
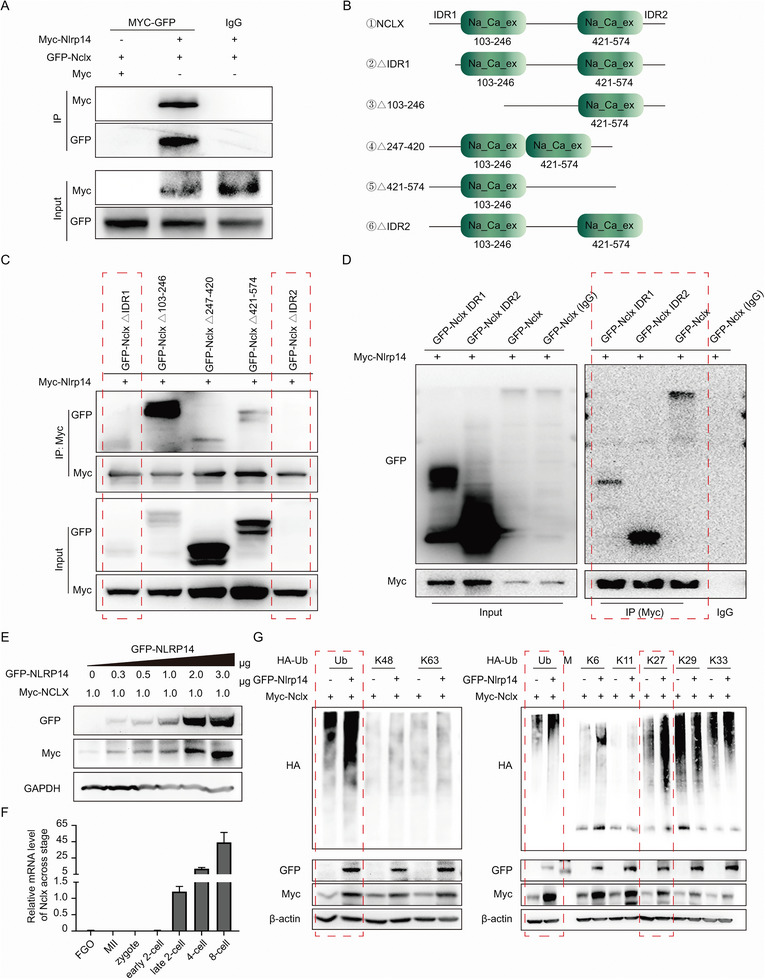
NLRP14 maintained the stability of NCLX by regulating its K27 ubiquitination. A) Interaction between NLRP14 and NCLX was confirmed by immunoprecipitation. myc‐tag, myc‐tagged mouse NCLX, and GFP‐tagged mouse NLRP14 were expressed in HEK293T cells as indicated for 48 h, and then co‐IP (myc‐GFP) and Western blot analysis for NCLX and NLRP14. B) Schematic of mouse NCLX truncation mutants. C) Interaction between NLRP14 and truncated NCLX was confirmed by immunoprecipitation. GFP‐tagged truncated mouse NCLX and myc‐tagged mouse NLRP14 were expressed in HEK293T cells as indicated for 48 h, and then co‐IP and Western blot analysis for NCLX and NLRP14. D. Interaction between NLRP14 and NCLX‐IDR was confirmed by immunoprecipitation. GFP‐tagged mouse NCLX‐IDR and myc‐tagged mouse NLRP14 were expressed in HEK293T cells as indicated for 48 h, and then co‐IP and Western blot analysis for NCLX and NLRP14. E. GFP‐tagged mouse NLRP14 and myc‐tagged mouse NCLX were expressed in HEK293T cells as indicated for 48 h. The amount of GFP‐tagged mouse NLRP14 plasmid is gradient increased as indicated. F. qRT–PCR showing the relative levels of Nclx transcripts in GV oocytes, MII oocytes, and zygotes. G. GFP‐tagged mouse NLRP14, myc‐tagged mouse NCLX and HA‐tagged ubiquitin or ubiquitin mutants were expressed in HEK293T as indicated for 48 h, and then co‐IP and western blot analysis for the ubiquitination of myc‐tagged mouse NCLX.

The results so far indicated that NLRP14 was required for NCLX stability. We next set out to investigate a possible interplay between NLRP14 and NCLX proteins. To investigate this question, we constructed plasmids expressing GFP‐NCLX, Myc‐NCLX, and a series of truncated NCLX mutants. We detected the interaction between NLRP14 and NCLX proteins by Co‐immunoprecipitation (Co‐IP) in cells co‐expressing the two proteins (myc‐NLRP14 and GFP‐NCLX), confirming that NLRP14 does interact with NCLX (**Figure** **8**A). Then we asked which domain of NCLX was required for interaction with NLRP14. Through Co‐IP experiments, we found that truncated NCLX including NCLX^△103‐246^, NCLX^△247‐420,^ and NCLX^△421‐574^ could still interplay with NLRP14 proteins (Figure 8B,C). However, IDRs mutants of NCLX in N‐terminal and C‐terminal were not expressed, which indicated that the IDRs domain is important to NCLX stability (Figure 8C). In addition, in order to further analyze whether the intrinsically disordered regions (IDRs) domain was necessary for the interaction between NLRP14 and NCLX, we constructed plasmids expressing GFP‐NCLX‐IDR1 (the N‐terminal) and GFP‐NCLX‐IDR2 (the C‐terminal). Notably, Co‐IP analyses showed that both IDR1 and IDR2 could directly interact with NLRP14 (Figure 8D). This result indicated the importance of IDRs domain for the interaction of NCLX to NLRP14. Intriguingly, we noticed that co‐expression of NLRP14 with NCLX often led to increased protein levels of NCLX. We asked whether NLRP14 could increase NCLX levels in a dose‐dependent manner. As shown in Figure 8E, co‐expressed Myc‐NCLX with an increasing amount of GFP‐NLRP14 in HEK293T cells indeed led to the increase of NCLX level. To exclude the possibility that the loss in NCLX protein was caused by reduced expression of the *Nclx* mRNA, we used RT‐PCR to analyze the *Nclx* mRNA and found that the abundance of *Nclx* mRNA was absent until late 2‐cell after zygotic gene activation (Figure 8F). This result indicated that the expression of NCLX might be mainly regulated by posttranslational modifications. Then we co‐expressed GFP‐NLRP14 and the Myc‐NCLX with HA‐ubiquitin (HA‐Ub) to determine whether NLRP14 stabilized NCLX by regulating its ubiquitination modification. GFP‐NLRP14 overexpression significantly increased Myc‐NCLX ubiquitination compared with the GFP‐NLRP14 absent group (Figure 8G). We co‐expressed HA‐tagged ubiquitin mutants with Myc‐NCLX in the absence or presence of GFP‐NLRP14 to characterize the possible linkage of ubiquitin chains on NCLX. Meanwhile, immunoprecipitation assays showed that NLRP14 predominantly mediated the K27‐linked ubiquitination (all others were replaced with arginine), but there was no obvious change at levels with ubiquitin containing K48, K63, K6, K11, K29, or K33 alone (Figure 8G). Notably, K27‐linked chain has been shown to slow down degradation by the proteasome.^[^ ^14^ ^]^ Taken together, these data show that the IDRs domain of NCLX were sufficient for its interaction with NLRP14 and subsequently modified with K27‐linked ubiquitination.

## 3 Discussion

The early events of embryonic development in mammals are mainly controlled by maternal effectors, which are encoded by maternal effect genes, accumulated, and stored during oogenesis. The importance of maternal factors for quality control of mammalian oocytes is self‐evident. However, our understanding of the maternal effect genes involved in female fertility and early embryo development is very limited in mammals. In this study, we demonstrate that NLRP14 acts as a safeguard of Ca^2+^ homeostasis by interacting with NCLX IDRs domain and regulating its K27‐linked ubiquitination.

NLRP14 female KO mice were completely sterile and exhibited 2‐cell developmental arrest. Yan et al. recently reported that UHRF1 and DNMT1 shifted from cytoplasmic localization to nuclear localization in zygotes that lacked maternal NLRP14, which hindered the occurrence of passive DNA demethylation associated with DNA replication and resulted in significant higher DNA methylation levels in the paternal genome than control embryos.^[^ ^10^ ^]^ Notably, DNA methylation can be normally established in *Nlrp14*‐deficient oocytes. However, through a spindle transfer assay, we found that *Nlrp14^mNull^* embryo development arrest was mainly caused by cytoplasmic defects, suggesting that NLRP14 plays a more important function in the cytoplasm. The protein level of UHRF1 is significantly decreased (nearly loss) in oocytes lacking NLRP14. Through systematic screening of cytoplasm‐related indicators, we found that the ATP content in the cytoplasm was significantly reduced, and the mitochondrial morphology and dynamics were severely damaged in *Nlrp14*‐deficient oocytes. Of note is the failure of parthenogenetic activation in *Nlrp14*‐deficient oocytes, with severe abnormalities in calcium oscillation patterns and very high cytoplasmic calcium concentrations. Intriguingly, loss of maternal UHRF1 caused the obvious decrease of NLRP14 protein level. In addition, NLRP14 interacted with UHRF1. Surprisingly, oocytes lacking maternal UHRF1 also maintained a high intracellular Ca^2+^ concentration and displayed an aberrant [Ca^2+^]i oscillations pattern upon parthenogenetic activation. The present study reveals a novel function of UHRF1 in calcium homeostasis beyond a DNA methylation regulator. The major and most surprising finding in our study is that loss of NLRP14 or UHRF1 would disrupt calcium homeostasis and thus oocyte activation and early embryonic development failure. It will be very interesting to determine whether other proteins are involved in the formation of this complex, providing detailed molecular and functional insights into this maternal module and how it orchestrates calcium homeostasis in mouse oocytes.

Another interesting aspect of our study is that NCLX is the only mitochondrial Ca^2+^ transport channel significantly affected in *Nlrp14^mNull^* oocytes. *Nclx* transcript was undetectable in oocytes, which suggests that NCLX function in oocytes is mainly regulated at the protein level. Our data revealed that loss of maternal NLRP14 caused a striking decrease of NCLX, which disrupts the [Ca^2+^]i and [Ca^2+^]m in oocytes. In addition, our study showed that NLRP14 regulates NCLX K27‐linked ubiquitination through an unknown E3 ligase. Future studies need to identify the underlying mechanism by which NLRP14 regulated K27‐linked ubiquitination of NCLX.

Ca^2+^ is of central importance for a series of cellular processes including energy generation, chromosome segregation, pronucleus formation, and gene expression. It has been demonstrated that calcium homeostasis is essential for oocyte activation and embryonic development.^[^ ^15^ ^]^ Once the sperm and oocyte are fused, the cytosolic Ca^2+^ rises, which is a prerequisite for successful fertilization and oocyte‐to‐embryo transition. [Ca^2+^]i oscillations last for several hours, which is essential for embryo development after fertilization. The regulatory mechanism of long‐lasting series of repetitive [Ca^2+^]i oscillations during oocyte activation is still a mystery.^[^ ^5^ ^]^ Moreover, mitochondria possess a series of Ca^2+^ transport influx and efflux channels to buffer Ca^2+^ in the cytoplasm.^[^ ^7^ ^]^ It is worth noting that [Ca^2+^]m must be finely regulated because excessive Ca^2+^ could disturb oxidative phosphorylation (OXPHOS) in mitochondria, namely, mitochondrial Ca^2+^ uptake and release must be balanced in the homeostatic condition.^[^ ^7^ ^]^ Yet, *Mcu* null female mice in a mixed genetic background are fertile with little or no energy crisis in the heart.^[^ ^6^, ^16^ ^]^ An emerging concept from a series of studies suggests that a mitochondrial Na^+^/Ca^2+^ exchanger (NCLX, also named as Slc8b1) is essential for Ca^2+^ homeostasis.^[^ ^6^, ^17^ ^]^ Furthermore, mitochondrial dynamics, including fission, fusion, motility, and morphology, play an even more critical role in the functional status of mitochondria and eukaryotic cells.^[^ ^18^ ^]^ In fact, female mice with oocytes deficient for mitochondrial fission are infertile.^[^ ^19^ ^]^ Mitochondrial architecture is strikingly different in oocytes compared with somatic cells, characterized by its smaller and rounder appearance in oocytes.^[^ ^20^ ^]^ Consistent with previous studies that calcium directly or indirectly regulates mitochondrial dynamics,^[^ ^21^ ^]^ *Nlrp14^mNull^* oocytes that are deficient in calcium homeostasis showed significant mitochondrial dysfunction such as being more prone to fusion than fission, restricted mitochondrial movement and lower mitochondrial oxidative capacity. Given that many mutations in genes encoding cytoplasmically localized proteins often cause abnormal oocyte‐to‐embryo transition, we can screen the Nlrp14 mutations in patients whose embryos are arrested in early cleavage, so as to provide possible guidance for diagnosis and treatment of such clinical cases.

Taken together, this study has systematically revealed, for the first time, that NLRP14 is an essential maternal factor that regulates oocyte Ca^2+^ homeostasis by interacting with NCLX IDRs domain and regulating its K27‐linked ubiquitination and that UHRF1 cooperates with NLRP14 to ensure the normal calcium homeostasis in mouse oocytes. Our study answered a fundamental and unanswered question on how maternal factors regulate calcium homeostasis in oocytes, especially during calcium oscillations.

## 4 Experimental Section

### Mice

Oligos encoding a single guide RNA (sgRNA) that targets the exon 3 of *Nlrp14* were inserted into px330 plasmid. Unique sgRNA sequence was chosen on the basis of the Genetic Perturbation Platform from the Broad Institute website. An *Nlrp14* targeting vector was constructed and microinjected with Cas9 mRNA and sgRNA into zygotes of C57BL/6 mice. The sgRNA was designed to target exon 3 of the endogenous mouse *Nlrp14* gene. The genotypes of mice including *Nlrp14* ^+/+^, *Nlrp14* ^+/−^and *Nlrp14* ^−/−^ were confirmed by DNA sequencing. *Nlrp14* ^+/+^ and *Nlrp14* ^+/−^ female mice were designated as “control”. *Nlrp14* ^−/−^ female mice were designated as “*Nlrp14* ^mNull^”. The mutant mouse line was maintained on a mixed genetic background of C57BL/6 and CD1.

Generation of mice expressing 3xflag tagged NLRP14 was performed by Beijing Biocytogen, China. Briefly, the tags including 3xflag, GFP, and iCreERT2 were inserted before stop codon of *Nlrp14* CDS using Crispr/Cas9. P2A and IRES sequence allow for co‐expression of Nlrp14‐3xflag, GFP, and iCreERT2 from a single transcript and independent translation. Cas9 mRNAs, gRNAs, and dsDNA donors were mixed for cytoplasmic injection into the mouse zygotes from C57BL/6 female mice. The F0 mice were genotyped with the PCR primer specific for inserted tags (Table S3, Supporting Information). The F0 Nlrp14 knock‐in (KI) mice were crossed with wild‐type C57BL/6 mice to generate F1 heterozygous KI mice. F1 heterozygous KI mice were crossed to generate F2 homozygous KI mice.

The previously described Stra8‐GFP:Cre knock‐in mice were generously provided by the Ming‐Han Tong lab.^[^ ^22^ ^]^ The GFP:Cre tag sequence was inserted before stop codon of Stra8 CDS, which was linked by a P2A peptide sequence to allow co‐expression of Stra8 and GFP:Cre. The previously described *Uhrf1^flox/flox^* mice^[^ ^11c^ ^]^ (Shanghai Research Center for Model Organisms) were maintained on the C57BL/6J (B6) background. The oocyte‐specific knockout mice with the deletion of *Uhrf1* exon 4 were generated by crossing *Uhrf1* ^flox/flox^ mice with Stra8‐GFP:Cre knock‐in mice. *Uhrf1^flox/△^; Stra8‐GFP:Cre+* females (designated as *Uhrf1^flox/△^;SKO*).

All animal studies were conducted in accordance with and approved of the guidelines of the Animal Care and Use Committee of the Institute of Zoology at the Chinese Academy of Sciences. Mice were killed under standard protocols, and all efforts were made to minimize suffering.

### Fertility Assessment, Natural Ovulation, and Superovulation Analysis

To assess the reproductive activity, six individually housed control or mutant female mice were mated with a male mouse validated to be fertile at the age of 6 weeks, respectively. The number of pups was recorded up to 6 months. For the natural ovulation assay, 6–9‐week‐old adult female mice in estrus were mated with fertile males overnight. The next day in the morning, successful mating was confirmed by the presence of vaginal plugs. Zygotes were collected from oviducts and counted. For the superovulation analysis, 6–9‐week‐old adult female mice were subjected to intraperitoneal injection with 10 IU of PMSG. After 48 h, 10 IU of hCG was injected to promote ovulation. The cumulus–oocyte complexes derived from the oviducts were collected at 13 h of hCG treatment. After a 2–3 min treatment with 0.5 mg mL^−1^ hyaluronidase in the M2 medium, MII oocytes were collected, counted, and then analyzed.

### Antibodies

Polyclonal rabbit antibody against NLRP14 was generated, and the specificity of NLRP14 antibody was verified by Western blot. Polyclonal rabbit antibody against STELLA was generously provided by the Bing Zhu lab. SCMC components‐related antibodies including MATER mouse antibody (140 kDa), TLE6 mouse antibody (70 kDa), FILIA sheep antibody (55 kDa), ZBED3 rabbit antibody (36 kDa), and FLOPED rabbit antibody (19 kDa) were generous gifts from the Lei Li lab. Commercial antibodies used in this study include: anti‐Flag (for IP and WB, Sigma, F1804), anti‐Flag (for IF, ABclonal, AE005), anti‐FLAG(R) M2 Magnetic Beads (Sigma, M8823), anti‐tubulin‐F488 (Thermo, 322588), anti‐Centromere Protein Antibody (for IF, Antibodies Inc, 15–234), anti‐mUHRF1 (for WB and IF, Santa Cruz sc‐373750 (H‐8), anti‐DNMT1 (for WB, MyBioSource, MBS179160), anti‐Drp1 (for WB, abcam, ab184247), anti‐NCLX (for WB, HUABIO, ER1901‐93), anti‐ COX IV (gplink, P01L083), anti‐LETM1 (ABclonal, A3945), anti‐HA (for IP and WB, Easybio, BE2007‐100), anti‐HA (for ubiquitin assay, cst, 3724), anti‐myc (for IP, Sigma, M4439), anti‐myc (for WB, Proteintech, 9E1), anti‐GFP (for IP and WB, Abcam, ab290), anti‐β‐actin (Easybio, BE0021‐100), anti‐GAPDH (ABclonal, AC033), anti‐5hmc (Active Motif, 39792), anti‐γ‐H2AX (for IF, cst, 9718S), and the following secondary antibodies were used: goat anti‐rabbit IgG(H + L) Alexa Fluor 488 (Invitrogen; A‐11008; 1:1000); goat anti‐mouse IgG(H + L) Alexa Fluor 488 (Invitrogen; A‐11001; 1:1000); goat anti‐rabbit IgG(H + L) Alexa Fluor 594 (Invitrogen; A‐11012; 1:1000); goat anti‐mouse IgG(H + L) Alexa Fluor 594 (Invitrogen; R37121; 1:1000).

### Cell Culture and Transfection

HEK293T cells were cultured in DMEM (C11330500BT, Gibco) supplemented with 10 FBS (ST30‐3302, PAN). Six‐well cultures were incubated at 37 °C, 5% CO_2_ in a humidified incubator. Cell density ranged between 70–80% confluency on the day of transfection. Then growth medium was removed from cells and replaced with 2 mL of complete growth medium. For each well of cells to be transfected, 4 µg of plasmid DNA and 8 µL of P3000 Reagent were diluted in 125 µL Opti‐MEM. For each well of cells, 8 µL of Lipofectamine 3000 Reagent was diluted with 125 µL Opti‐MEM. Diluted DNA was added to each tube of diluted Lipofectamine 3000 Reagent (1:1 ratio), mixed gently, and incubated for 10–15 min at room temperature to form DNA‐ Lipofectamine LTX Reagent complexes. DNA‐lipid complex was added to cells. After 5–12 h, the growth medium was removed from cells and replaced with 2 mL of complete growth medium.

### Parthenogenetic Activation and Real‐Time Recording of [Ca^2+^]i and [Ca^2+^]m Changes

For SrCl_2_‐induced activation in Ca^2+^‐free CZB, super‐ovulated metaphase‐II oocytes were washed three times in an M2 medium and then cultured in an activation medium (Ca^2+^‐free CZB containing 10 mM SrCl_2_) for 6 h. [Ca^2+^]i oscillations regulation were confirmed by mitochondrial activity during oocyte activation. [Ca^2+^]i oscillations were observed by staining with 2 µM Fluo‐4 AM or 2 µM Fluo‐8 AM (488 nm 132 excitation, 525 nm emission) in the partheno‐activation system. To detect [Ca^2+^]m dynamic changes, Mt‐GCaMP6s was introduced to obtain the patterns of mitochondrial matrix Ca^2+^ dynamic changes. Real‐time images were obtained using a time‐lapse confocal laser microscope (UltraVIEW‐VoX; PerkinElmer, MA, USA).

### Immunofluorescence Analysis and Chromosome Spread

Oocytes for immunofluorescence staining were fixed in 4% paraformaldehyde (PFA) in PBS for 30 min at room temperature. Then the oocytes were transferred to membrane permeabilization solution (0.5% Triton X‐100) for 20 min and blocking buffer (1% BSA in PBS) for at least 1 h. The oocytes were incubated overnight at 4 °C with the antibodies at appropriate dilutions. Then the oocytes were mounted on glass slides and examined with a laser scanning confocal microscope (Zeiss LSM 780 META, Germany). For chromosome spreads, the oocytes were first freed of the zona pellucida by acid Tyrode's solution (Sigma‐Aldrich). After a brief recovery in an M2 medium, the oocytes were transferred onto glass slides and fixed in a solution of 1% paraformaldehyde in distilled H_2_O (pH 9.2) containing 0.15% Triton X‐100 and 3 mM dithiothreitol. DNA on the slides was stained with DAPI and slides were mounted for observation by immunofluorescence microscopy. The fluorescent signal intensity was quantified with Image J.

### Real‐Time Recording of Mitochondrial Dynamics During Meiotic Maturation of Oocytes

Activated mitochondria were imaged with 0.1 µM MitoTracker (Invitrogen, M7512, China), a cell‐permeable potential‐sensitive fluorescent mitochondrial dye emitting in the red (561 nm excitation, 590 nm emission) channel, which had a high affinity with higher potential mitochondria, by using a time‐lapse confocal laser microscope (UltraVIEW‐VoX; PerkinElmer, USA). The GV oocyte meiotic maturation was conducted by incubation in an M2 medium (M7167, Sigma‐Aldrich, USA) containing 0.1 µM MitoTracker.

### Measurement of Cytoplasmic ATP Content in Oocytes

The measurement was performed using a Berthold Lumat LB 9501 luminometer (Berthold Technologies, Bad Wildbad, Germany) and a commercial assay kit based on the luciferin–luciferase reaction (Cat #S0026; Beyotime, China) following the manufacturer's recommendations. A standard curve with different concentrations from 0 nmol to 1 mmol was generated before the detection of ATP content in oocytes. For each experiment, twenty‐five oocytes were loaded in 200 µL PCR tubes and then stored at −80 °C until measurement of ATP content. The ATP content was finally calculated using the formula derived from the linear regression of the standard curve.

### Immunofluorescence

The method for immunofluorescent staining of CGs was performed according to a previous study.^[^ ^23^ ^]^ Briefly, oocytes were treated with a Tyrode solution for 1 min to remove the zona pellucida. Oocytes without zona pellucida were fixed with 4% PFA in PBS buffer for 30 min at room temperature. After being permeabilized with 0.1% Triton X‐100 for 20 min, they were then blocked in 1% BSA‐supplemented PBS for 1 h at room temperature, and then cultured with 1:200 lens culinaris (LCA)‐FITC for 1 h at room temperature. Oocytes were washed three times (5 min each time) in PBS buffer. The DNA was stained in the final incubation step for 15 min with DAPI. Finally, oocytes were mounted on glass slides and viewed under a confocal laser scanning microscope (Zeiss LSM 880). The methods for immunofluorescent staining of 5hmc were performed according to a previous study.^[^ ^24^ ^]^ Zygotes were fixed with 4% PFA in PBS buffer for 30 min at room temperature. After being permeabilized with 0.1% Triton X‐100 for 20 min, they were washed with PBS buffer containing 10 mg mL^−1^ BSA (PBS/BSA). Then these zygotes were treated with 4N HCl for 10 min and transferred into 0.1 m Tris‐HCl (pH 8.0) for 20 min to neutralize. Then the zygotes were incubated with PBS/BSA overnight. Anti‐5hmC (1/400: Active Motif) antibodies diluted in PBS/BSA were incubated for 1 h at room temperature. Then the zygotes were washed three times (5 min each time) in PBS/BSA, and incubated with corresponding fluorescent secondary antibodies for 1 h at room temperature, followed by incubation with Hoechst 33342 for 20 min. These cells were mounted on glass slides and analyzed using a laser‐scanning confocal microscope (Zeiss LSM 880).

### Transmission Electron Microscopy

The overall processes of biological sample preparation for transmission electron microscopy were as follows. About 60 oocytes were placed on a dish filled with pre‐cooled glutaraldehyde; the samples were embedded in agar and cut into small pieces of ≈1 mm^3^ with a double‐sided blade, and then rinsed with 0.1 m PBS for three times, 15 min/time. 1% osmium acid was added for fixation for 1–1.5 h, and then the samples were rinsed with 0.1 m PBS for three times, 15 min/time. The samples were dehydrated with gradient acetone of 30%, 50%, 70%, 80%, 90%, 95%, and 100%, 10–15 min each, and then replaced with 100% acetone twice, 10 −15 min/time. Then the samples were soaked with gradient acetone: resin, and finally embedded in pure resin and polymerized for 12 h at 37 °C, 12 h at 45 °C, 24 h at 60 °C, or directly polymerized at 60 °C for 48 h. Ultra‐thin sections at 60 nm were prepared and stained with uranyl acetate‐lead citrate double dyeing method. Finally, electron microscope observation was performed with an F20 Field Emission Gun Transmission Electron Microscope.

### Spindle Transfer

MII oocytes super‐ovulated from BDF1 strain female mice (or *Nlrp14^mNull^* female mice) at the age of two months were used as spindle‐chromosome complex and cytoplast donors. The transfer of the spindle‐chromosome complex was performed using a piezo‐driven micromanipulator. Enucleation and transplantation of spindle‐chromosome complexes were performed in manipulation drops of M2 medium. Then the transferred spindle‐chromosome complexes were exposed to the hemagglutinating virus of Japan envelope (Cosmo Bio, ISK‐CF‐001‐EX) and transplanted into the sub‐zona pellucida space of enucleated recipient oocytes. About 30 min later, these reconstructed oocytes were parthenogenetically activated.

### Real‐Time Quantitative Polymerase Chain Reaction (qPCR) Analysis of Mitochondrial DNA (mtDNA) Content in Oocytes

The total amount of mtDNA copy per oocyte was determined using quantitative real‐time PCR (qPCR) procedure according to a previous study.^[^ ^25^ ^]^ The mouse mtDNA‐specific primers were: mtB6 forward: AACCTGGCACTGAGTCACCA and mtB6 reverse: GGGTCTGAGTGTATATATCATGAAGAGAAT.^[^ ^26^ ^]^ 10‐fold serial dilutions of purified plasmid standard DNA ranging from 10 copies to 100 000 000 copies of mtDNA per 1 µL were used to generate the standard curve. The amplification efficiency of the standard curve was calculated using the qPCR efficiency calculator from Roche. Briefly, a single oocyte was loaded in a PCR tube with 10 µL lysis buffer containing 0.12 mg mL^−1^ proteinase K and incubated at 55 °C for 2 h, then 95 °C for 10 min, and then 2 µL of sample was taken directly for qPCR analysis. The cycling conditions were as follows: an initial phase of 10 min at 95 °C followed by 40 cycles of 15 s at 95 °C and 1 min at 60 °C. The melting temperature was 72 °C for 1 min. Linear regression analysis of all standard curves for samples with copy numbers between 10^3^ and 10^8^ showed a correlation coefficient higher than 0.97. The standards of known mtDNA copy number (described above) were included during each reaction. Each oocyte measurement was performed in triplicates at least. At least fifteen MII oocytes from three mice were analyzed.

### Immunoprecipitation Assay

HEK293T cells were cultured in DMEM (C11330500BT, Gibco) supplemented with 10 FBS (ST30‐3302, PAN). Six‐well cultures were incubated at 37 °C, 5% CO_2_ in a humidified incubator. For co‐IP of exogenous proteins, HEK293T cells were transfected with the indicated plasmid (s). The cells were collected 48 h after transfection and lysed in IP Lysis buffer (Beyotime Biotechnology, P0013) containing 1× protease inhibitor cocktail for 30 min at 4 °C with occasional vortexing. The lysates were cleared by centrifugation at maximum speed for 20 min at 4 °C. Then the supernatant was transferred to a new tube and incubated with 1 µL corresponding primary antibody plus 20 µL protein A‐agarose beads as indicated. Incubated tubes were placed on a rotator at 4 °C for 4 h to overnight. Then the beads were washed six times in lysis buffer. The protein‐beads complexes were boiled with 1X SDS‐PAGE buffer at 95 °C for 5 min, and analyzed by SDS‐PAGE.

### LC–MS/MS Analysis Of Mouse Oocytes

For each sample, 50 MII oocytes were collected into a 1.5 ml EP tube. 50 µL 0.2% sodium deoxycholate was added to the oocytes for lysis on ice for 30 min. Subsequently, samples were reduced by 5 mM DL‐Dithiothreitol (Sigma, D9779) and alkylated with 10 mM iodoacetamide (Sigma, I1149). After digestion by Trypsin/Lys‐c Mix (Promega, V5071) overnight, the peptide solution was desalted using SOLAµ HRP SPE spin plates (Thermo Scientific, 60209‐001). After desalting, the eluent was dried in a vacuum evaporator. Samples were redissolved into 0.1% formic acid before use.

Samples were performed on a timsTOF Pro mass spectrometer (Bruker Daltonics) in dda‐PASEF mode with a 120‐min nonlinear LC gradient. MS and MS/MS spectra were recorded from *m*/*z* 100 to 1700 with 1/K0 range from 0.6 V·s cm^−2^ to 1.6 V·s cm^−2^. The ramp time was set to 100 ms while keeping the duty cycle fixed at 100%. A total cycle time of 1.16 s contained one MS1 scan and ten PASEF MSMS scans. MS/MS spectra were searched against the UniProtKB/Swiss‐Prot mouse database using Peaks Online X. The precursor tolerance was limited to 15 ppm and the fragment tolerance was set as 0.05 Da.

### IP‐MS

For each sample, fourteen ovaries were lysed in IP Lysis buffer (Beyotime Biotechnology, P0013) (20 mM Tris (pH 7.5), 150 mM NaCl, 1% Triton X‐100, 1 mM PMSF, 1 × Roche complete mini protease inhibitor cocktail, and 1 × Pierce phosphatase‐inhibitor cocktail) for 30 min at 4 °C with occasional vortexing. The lysates were cleared by centrifugation at maximum speed for 20 min at 4 °C. Then the supernatant was transferred to a new tube and incubated with anti‐FLAG M2 Magnetic Beads (Sigma, M8823) for 6 h to overnight. After extensive washing with IP lysis buffer, the protein‐beads complexes were transferred to a new 1.5 ml EP tube. These samples were eluted with 100 µL of glycine HCl (pH 2.5–3.0). The eluent was precipitated with 600 µL ice‐cold acetone for 6 h at −20 °C and then centrifuged at 16 000×g for 30 min. The precipitate was denatured with 8 m urea, reduced, and alkylated with 5 mM Tris(2‐carboxyethyl) phosphine hydrochloride (Thermo Scientific, PG82080) and 10 mM iodoacetamide. Digestion was performed with Trypsin/Lys‐c Mix. The resulting peptide was desalted and then dried in a vacuum evaporator. Samples were redissolved into 0.1% formic acid before use.

### Statistical Analysis

For each experiment, at least three replicates were performed. GraphPad Prism 8.02 (GraphPad Software) was employed to perform statistical analysis. The differences were assessed by unpaired Student's t‐test. Statistical significance was ascribed to *p*< 0.05. Graphs were presented as the mean standard error of the mean ± SEM.

## Conflict of Interest

The authors declare no conflict of interest.

## Author Contributions

Q.Y Sun and Z.H Li conceived the project. T.G. Meng designed the experimental scheme. T.G Meng wrote the manuscript and Q.Y Sun and H, Schatten reviewed and edit the manuscript. C Wong, Z.B Wang and X.H Ou Y.C Ouyang, and Z.M Han provided technical support. T.G Meng and J.N Guo performed most of the experiments. Y.K Yin designed *Nlrp14* knockout mice and Nlrp14 knockin mice. L Zhu performed LC–MS/MS and IP‐MS. T‐G M and J‐N G and L.Z. and Y.Y. contributed equally.

## Supporting information

Supporting Information

Supplemental Video 1

Supplemental Video 2

Supplemental Video 3

Supplemental Video 4

Supplemental Video 5

Supplemental Video 6

Supplemental Video 7

Supplemental Video 8

Supplemental Video 9

Supplemental Video 10

Supplemental Video 11

Supplemental Video 12

Supplemental Video 13

Supplemental Table 1

Supplemental Table 2

Supplemental Table 3

## Data Availability

The data that support the findings of this study are available from the corresponding author upon reasonable request.
