# Supplementary material for: NLRP14 Safeguards Calcium Homeostasis via Regulating the K27 Ubiquitination of Nclx in Oocyte‐to‐Embryo Transition
Source: Adv Sci (Weinh). 2023 Jul 26;10(27):2301940. doi: 10.1002/advs.202301940 (PMC10520637; doi:10.1002/advs.202301940)
Supplement: Supplementary file 1 — Supporting Information [file ADVS-10-2301940-s005.pdf]

ADVANCED SCIENCE
Open Access

Supporting Information

for *Adv. Sci.*, DOI 10.1002/advs.202301940

NLRP14 Safeguards Calcium Homeostasis via Regulating the K27 Ubiquitination of Nclx in Oocyte-to-Embryo Transition

Tie-Gang Meng, Jia-Ni Guo, Liu Zhu, Yike Yin, Feng Wang, Zhi-Ming Han, Lei Lei, Xue-Shan Ma, Yue Xue, Wei Yue, Xiao-Qing Nie, Zheng-Hui Zhao, Hong-Yong Zhang, Si-Min Sun, Ying-Chun Ouyang, Yi Hou, Heide Schatten, Zhenyu Ju, Xiang-Hong Ou*, Zhen-Bo Wang*, Catherine C. L. Wong*, Zhonghan Li* and Qing-Yuan Sun**

Supplementary Materials for

NLRP14 safeguards calcium homeostasis via regulating the K27 ubiquitination of NCLX in oocyte-to-embryo transition

Tie-Gang Meng et al.

Corresponding author: Qing-Yuan Sun, sunqy@gd2h.org.cn

This PDF file includes:

Figure S1 to S7

Other supplementary material for this manuscript includes:

Video 1 to 13

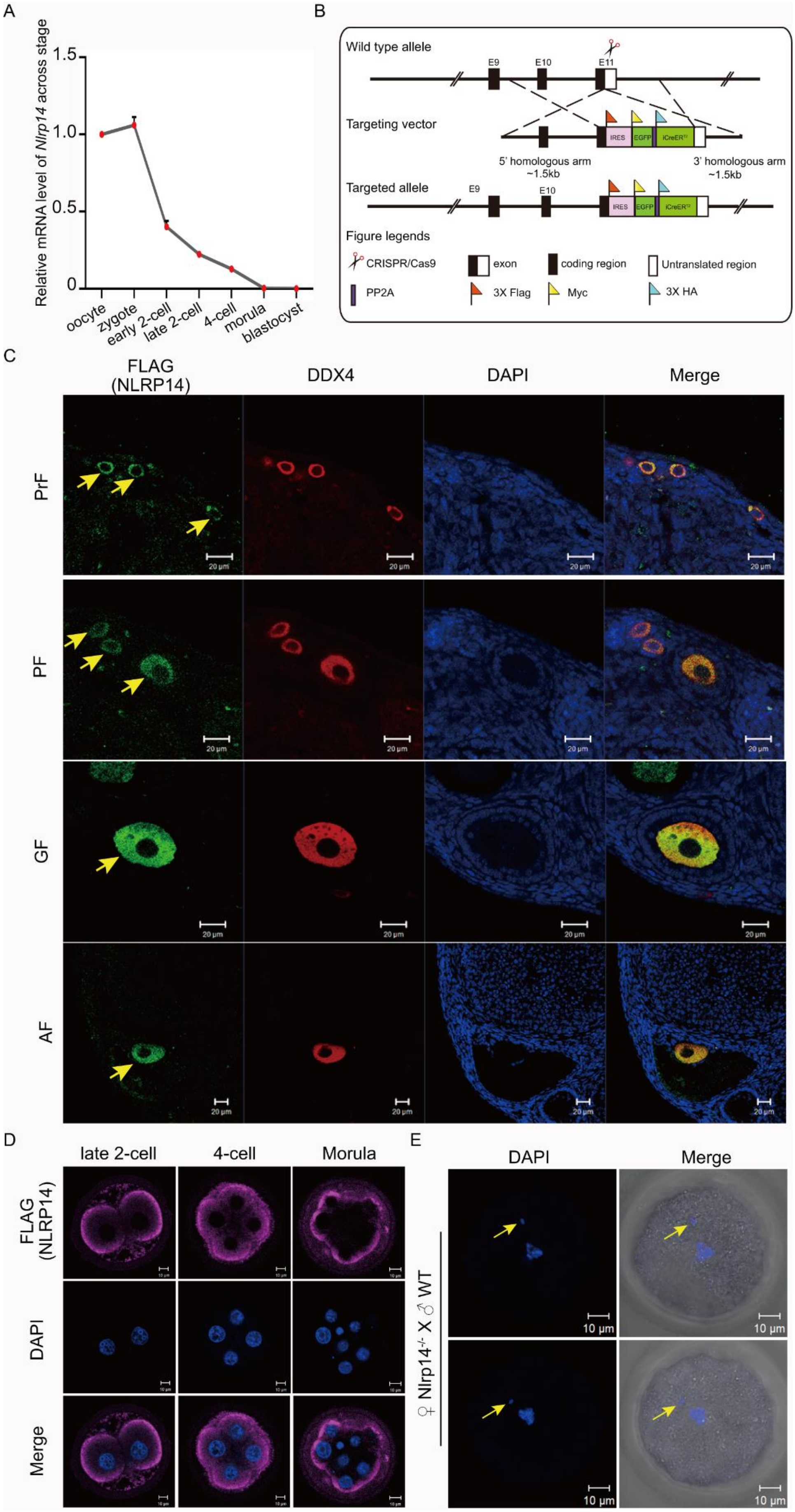

A
Relative mRNA level of Nlrp14 across stage
1.5
1.0
0.5
0
oocyte
zygote
early 2-cell
late 2-cell
4-cell
morula
blastocyst
B
Wild type allele
E9
E10
E11
Targeting vector
IRES
EGFP
iCreER T2
5' homologous arm
~1.5kb
3' homologous arm
~1.5kb
Targeted allele
Figure legends
CRISPR/Cas9
exon
coding region
Untranslated region
PP2A
3X Flag
Myc
3X HA
C
FLAG
(NLRP14)
DDX4
DAPI
Merge
PrF
PF
GF
AF
20 μm
D
late 2-cell
4-cell
Morula
FLAG
(NLRP14)
DAPI
Merge
E
DAPI
Merge
♀ Nlrp14-/- X ♂ WT
10 μm

Figure S1. NLRP14 is a maternal effect factor. **A**, *Nlrp14* mRNA expression in oocyte, zygote, early 2-cell, late 2-cell, 4-cell, morula and blastocyst embryos by quantitative RT-PCR. The level of *Nlrp14* mRNA in oocytes was set as control. **B**, Establishment of Nlrp14-3xflag knock-in mouse model, the tags including *3xflag*, GFP and iCreER^{T2} were inserted before stop codon of Nlrp14 CDS using Crispr/Cas9. P2A and IRES sequence allows for co-expression of Nlrp14-*3xflag*, GFP and iCreER^{T2} from a single transcript and independent translation. **C**, The expression and localization of NLRP14-FLAG (green) were detected in Nlrp14-3xflag knockin female ovary. Ovary sections were immunostained with an anti-FLAG antibody (green)， anti-DDX4 antibody (red) and DAPI (blue). **D**, Representative images of subcellular localization of NLRP14 in late 2-cell embryos, 4-cell embryos and morula. Embryos derived from Nlrp14-3xflag knockin female mice were immunolabeled with FLAG antibody (pink) and counterstained with DAPI (blue). **E**, Representative immunofluorescence staining images of oocytes that appear to be unfertilized are actually fertilized eggs. Yellow arrows indicate the sperm that could not form a pronucleus.

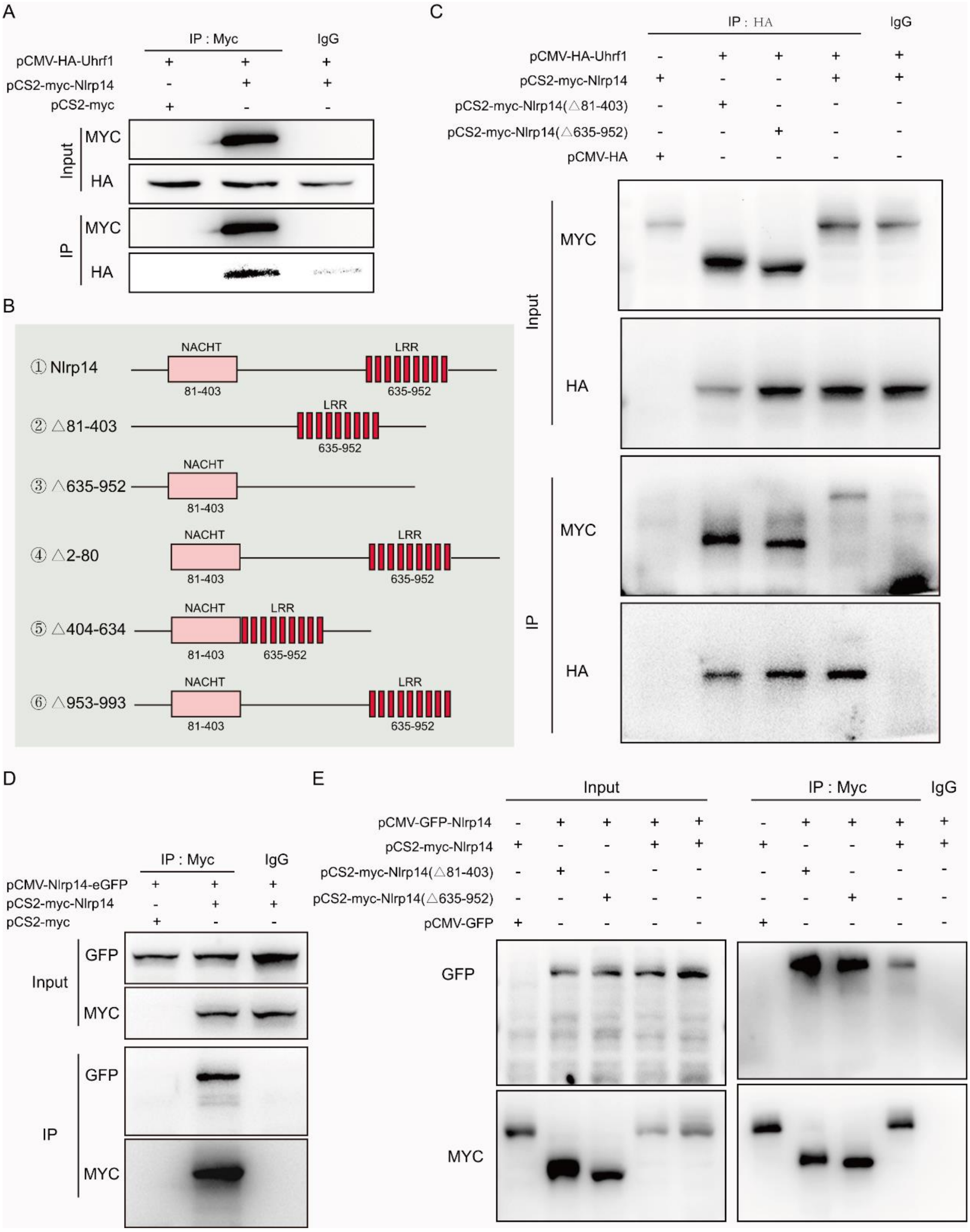

Figure S2. Specificity and interaction domain analysis for the UHRF1 and NLRP14 proteins. A, Interaction between NLRP14 and UHRF1 was confirmed by immunoprecipitation. MYC-tag, MYC-tagged mouse NLRP14 and HA-tagged mouse UHRF1 were expressed in HEK293T cells as indicated for 48h, and then co-IP (MYC-HA) and western blot analysis for NLRP14 and UHRF1. **B**, Schematic of mouse NLRP14 truncation mutants. **C**, HA-UHRF1 full length and truncation mutants of MYC-NLRP14 were expressed in HEK293T as indicated for 48h, and then co-IP (HA-

MYC) and western blot analysis for NLRP14 truncation mutants and UHRF1. **D**, NLRP14 can interact homotypically. MYC-tagged mouse NLRP14 and EGFP-tagged mouse NLRP14 were expressed in HEK293T cells as indicated for 48h, and then co-IP (MYC-EGFP) and Western blot analysis for NLRP14 with different tags. **E**, EGFP-NLRP14 full length and truncation mutants of MYC-NLRP14 were expressed in HEK293T as indicated for 48h, and then co-IP (MYC-EGFP) and Western blot analysis for NLRP14 truncation mutants and full length NLRP14.

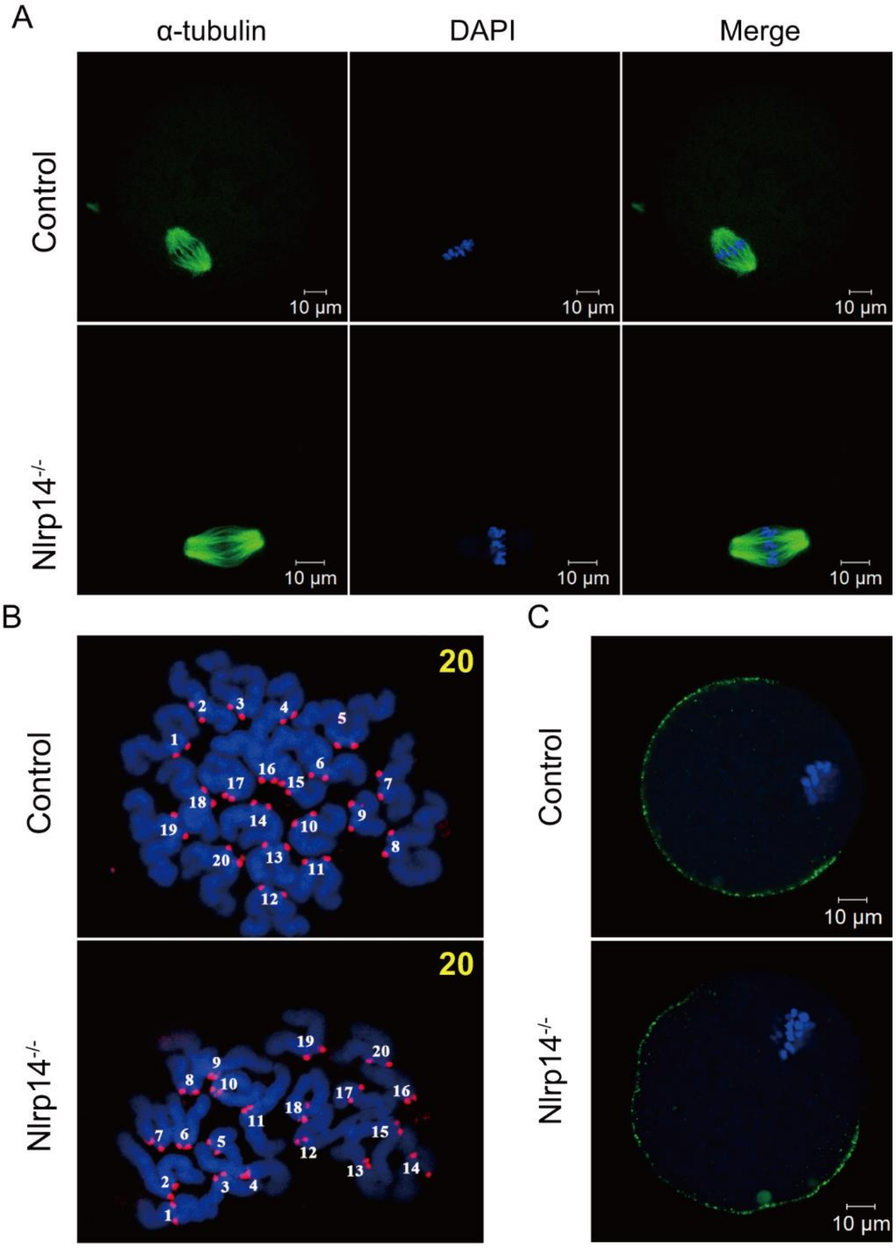

Figure S3. Analysis of the major events during meiosis A, Representative images of staining for DNA (red) and immunostaining for α-tubulin (green) showing normal spindle assembly in MII oocytes with indicated genotypes, respectively. **B**,

Chromosome spreading of metaphase-II oocytes immunostaining for DNA (blue) and ACA (red). **C**, Representative images of immunostaining for FITC-LCA, showing normal cortical granules (green) distribution of MII oocytes with indicated genotypes, respectively.

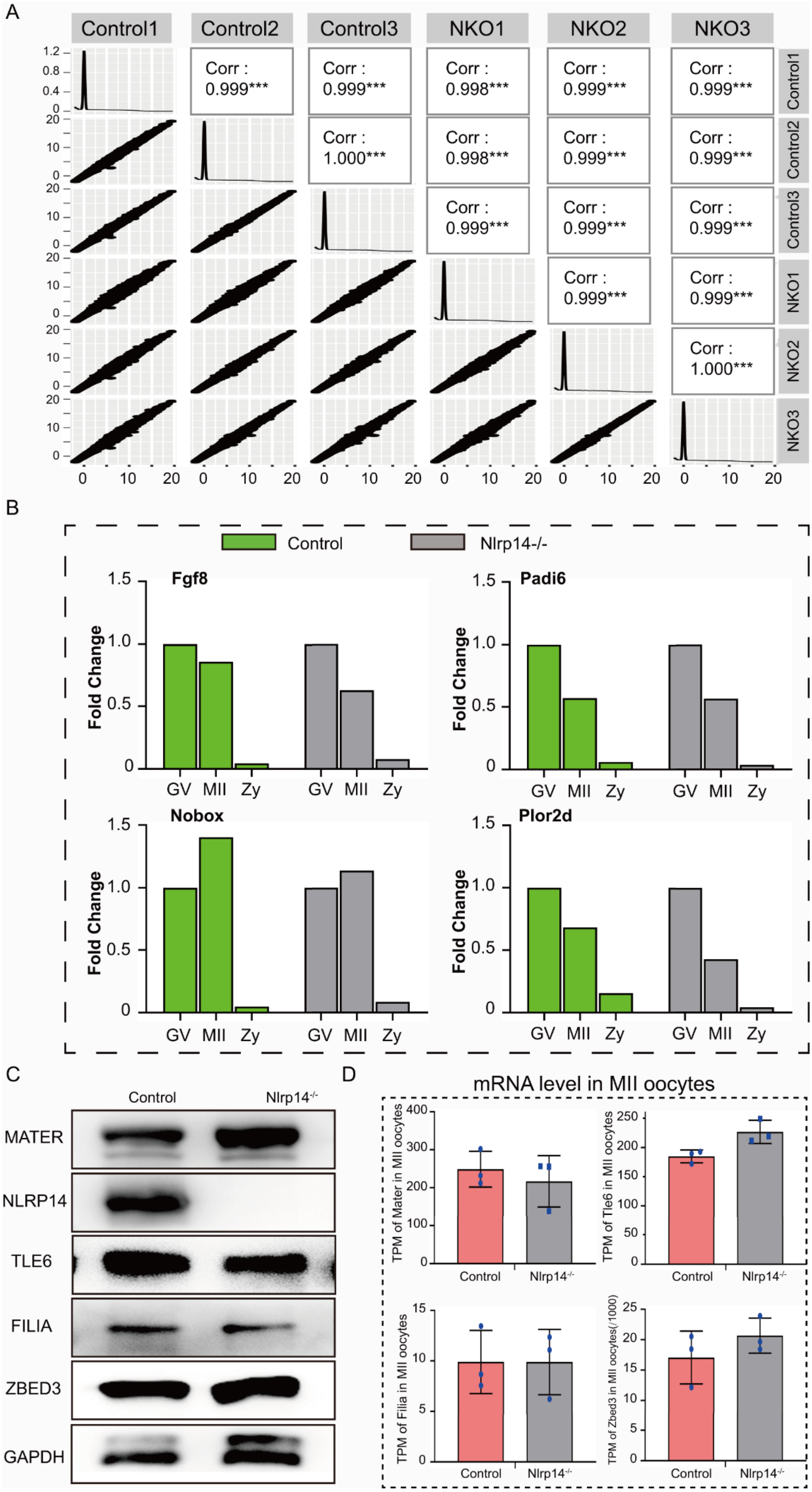
A
Control1
Control2
Control3
NKO1
NKO2
NKO3
Corr : 0.999***
Corr : 0.999***
Corr : 0.998***
Corr : 0.999***
Corr : 0.999***
Corr : 1.000***
Corr : 0.998***
Corr : 0.999***
Corr : 0.999***
Corr : 0.999***
Corr : 0.999***
Corr : 0.999***
Corr : 0.999***
Corr : 0.999***
Corr : 1.000***
B
Control
Nlrp14-/-
Fgf8
Padi6
Nobox
Plor2d
Fold Change
GV MII Zy GV MII Zy
C
Control
Nlrp14-/-
MATER
NLRP14
TLE6
FILIA
ZBED3
GAPDH
D
mRNA level in MII oocytes
TPM of Mater in MII oocytes
TPM of Tle6 in MII oocytes
TPM of Filia in MII oocytes
TPM of Zbed3 in MII oocytes(/1000)
Control
Nlrp14-/-

Figure S4. Analysis of maternal mRNA and SCMC complex. A, Pearson correlation analysis between all biological RNA-seq samples. Control indicates the *Nlrp14*$^{+/-}$ MII oocytes sample, NKO indicates the *Nlrp14*$^{-/-}$ MII oocytes sample. **B**, qRT–PCR showing the relative levels of the indicated transcripts in GV oocytes, MII oocytes and zygotes of control and *Nlrp14*^{mNull} female mice. **C,** Immunoblotting analyses of identified SCMC components in control and *Nlrp14*^{mNull} oocytes. **D,** The mRNA level of identified SCMC components in control and *Nlrp14*^{mNull} MII oocyte.

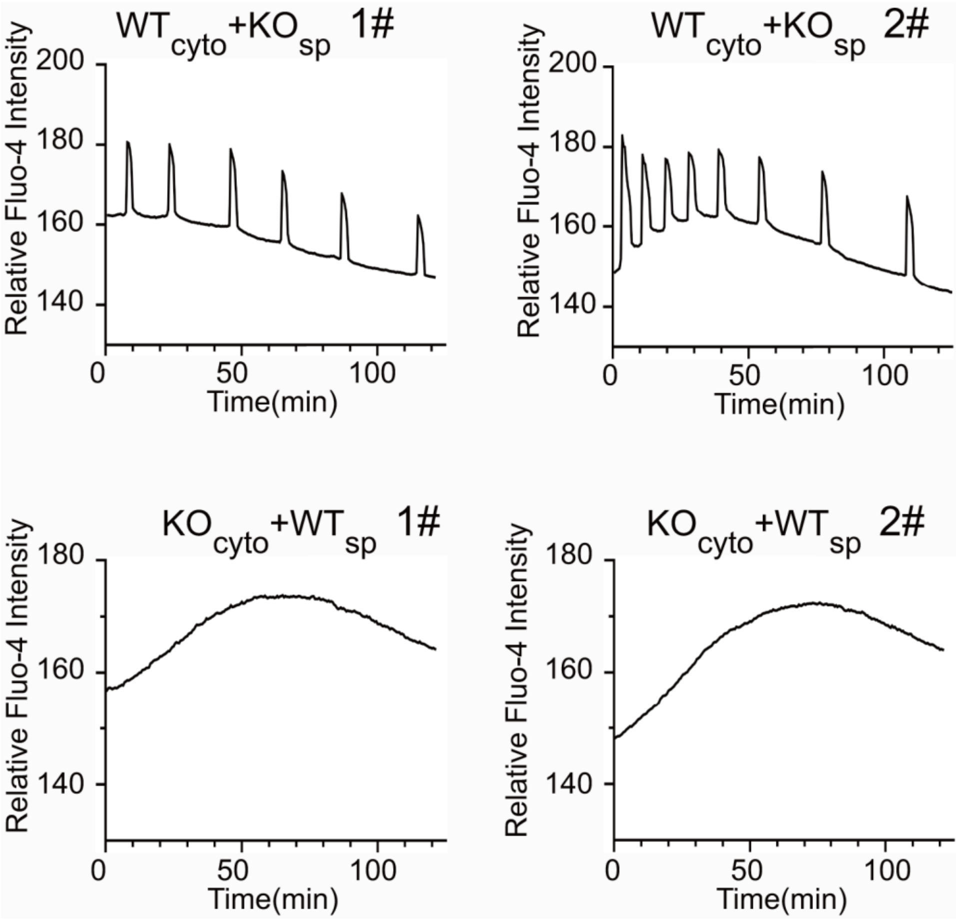

Figure S5. Ablation of maternal NLRP14 caused the failure of the [Ca^{2+}]i oscillations induced by parthenogenetic activation. [Ca^{2+}]i oscillation patterns after parthenogenetic activation of hybrid oocytes with indicated genotypes, respectively.

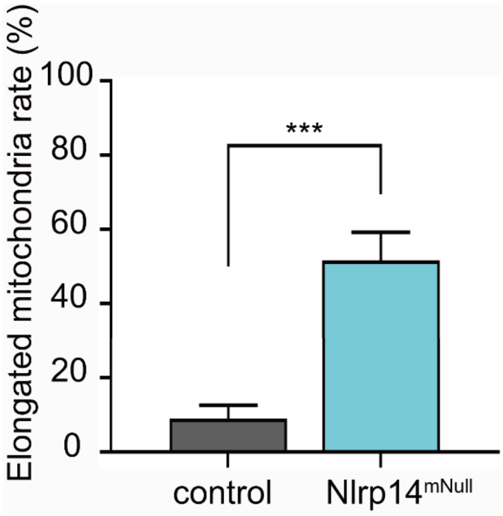

Figure S6. Mitochondria in Nlrp14mNull oocytes were elongated.

The rate of enlongated mitochondria in mouse oocytes with the indicated genotypes, respectively. Data are the mean ± SEM (n = 20).

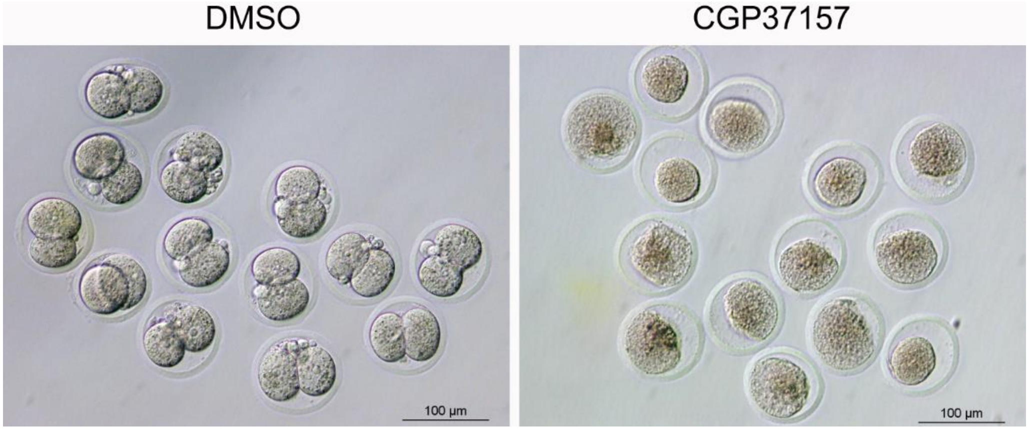

Figure S7. NCLX inhibition by CGP37157 caused oocyte death during PA. Oocytes treated with NCLX-specific inhibitor CGP37157. Untreated control group in addition to DMSO vehicle. Representative images of parthenogenetic activated embryos with the indicated treatment at day2, respectively.

Video 1. mitochondrial Ca^{2+} concentration ($[Ca^{2+}]m$) dynamic changes

during PA in control oocytes during PA

Video 2. mitochondrial Ca^{2+} concentration ($[Ca^{2+}]m$) dynamic changes in Nlrp14 ko oocytes during PA

Video 3. cytoplasmic Ca^{2+} concentration ($[Ca^{2+}]i$) in control oocytes during PA

Video 4. cytoplasmic Ca^{2+} concentration ($[Ca^{2+}]i$) in Nlrp14 ko oocytes during PA

Video 5. MitoTracker staining in control oocytes during meiotic maturation

Video 6. MitoTracker staining in Nlrp14 ko oocytes during meiotic maturation

Video 7. MitoTracker staining in control oocytes during meiotic maturation

Video 8. MitoTracker staining in Nlrp14 ko oocytes during meiotic maturation

Video 9. Cytoplasmic Ca^{2+} concentration ($[Ca^{2+}]i$) in control oocytes during PA

Video 10. Cytoplasmic Ca^{2+} concentration ($[Ca^{2+}]i$) in Uhrf1-sko #1 oocytes during PA

Video 11. Cytoplasmic Ca^{2+} concentration ($[Ca^{2+}]i$) in Uhrf1-sko #2 oocytes during PA

Video 12. Mitochondrial Ca^{2+} concentration ($[Ca^{2+}]m$) dynamic in control oocytes changes during PA

Video 13. Mitochondrial Ca^{2+} concentration ($[Ca^{2+}]m$) dynamic in Nlrp14 ko oocytes changes during PA
